# Only One Percent of Important Shark and Ray Areas in the Western Indian Ocean Are Fully Protected From Fishing Pressure

**DOI:** 10.1002/ece3.72690

**Published:** 2026-01-11

**Authors:** Jesse E. M. Cochran, Ryan Charles, Andrew J. Temple, Peter M. Kyne, Emiliano García‐Rodríguez, Adriana Gonzalez‐Pestana, Amanda Batlle‐Morera, Théophile L. Mouton, Asia O. Armstrong, Christoph A. Rohner, Darren J. Coker, Royale S. Hardenstine, Alexander Kattan, Ashlie J. McIvor, Viktor Nunes Peinemann, Kaitlyn A. O'Toole, Lea Palm, Eloise B. Richardson, Kalli Valappil Akhilesh, Haleh Ali Abedi, Reem K. Almealla, Dareen Almojil, Samantha Andrzejaczek, Arzucan N. Askin, Avik A. Banerjee, Hamid R. Bargahi, Alissa J. Barnes, Svetlana Barteneva‐Vitry, Siamak Behzadi, Aymeric Bein, Rhett H. Bennett, Filippo Bocchi, Ginevra Boldrocchi, Gill T. Braulik, Camrin D. Braun, Eleanor Brighton, Frances K. P. Budd, Robert W. Bullock, Clara Canovas Perez, Aaron B. Carlisle, Michelle Carpenter, Taylor K. Chapple, Isabel Chaúca, Geremy Cliff, Estelle Crochelet, Nakia Cullain, David J. Curnick, Ryan Daly, Leigh de Necker, Stella Diamant, Giulia F. A. Donati, David A. Ebert, Ehab Eid, Igbal S. Elhassa, Chantel Elston, Bernadine I. Everett, Mahmoud M. S. Farrag, Nico Fassbender, Sean T. Fennessy, Stela M. C. Fernando, Brittany Finucci, Anna L. Flam, Peter Gausman, Arnault R. G. Gauthier, Giri Bhavan Sreekanth, Trisha Gupta, Meral Hafeez, Badrú N. Hagy, Jessica L. A. Haines, Joanna L. Harris, Jessica Harvey‐Carroll, Tessa N. Hempson, Simon T. Hilbourne, Hua Hsun Hsu, Nor D. Ibrahim, David M. P. Jacoby, Sébastien Jaquemet, Idrees Babu K K, Divya Karnad, Boaz Kaunda‐Arara, Shoba J. Kizhakudan, Alison A. Kock, Anna Koester, Bigeyo N. Kuboja, Baraka L. Kuguru, James S. E. Lea, Omar Mahadalle, Hashim Manjebrayakath, Christophe Mason‐Parker, Daniel Mateos‐Molina, Muktha Menon, Alec B. M. Moore, Johann Mourier, Taryn S. Murra, Ajay D. Nakhawa, Nadeem Nazurally, Lauren E. Nelso, John E. G. Nevill, Jennifer M. Olbers, Raquel L. Ostrovski, Lauren R. Peel, Nathan Perisic, Bradley Peterson, Simon J. Pierce, Simon J. Pittman, Shikha Rahangdale, Joshua Rambahiniarison, Ali Reza Rastgoo, Mohsen Rezaie‐Atagholipour, David P. Robinson, Melita A. Samoilys, Tamaryn J. Sawers, Brittney J. Scannell, Jennifer V. Schmidt, Isabel M. Silva, Luis Silva, Jadiyde Solonomenjanahary, Julia L. Y. Spaet, Guy M. W. Stevens, Elspeth M. Strike, Sujitha Thomas, David van Beuningen, Stephanie K. Venables, Lennart Vossgaetter, Ornella C. Weideli, Ivor D. Williams, Collin T. Williams, Andrew J. Willson, Livi Wilson, Irthisham H. Zareer, Kaitlyn M. Zerr, Michael L. Berumen, Rima W. Jabado

**Affiliations:** ^1^ Reef Ecology Lab, Division of Biological and Environmental Science and Engineering King Abdullah University of Science and Technology Thuwal Saudi Arabia; ^2^ International Union for Conservation of Nature Species Survival Commission Shark Specialist Group Dubai UAE; ^3^ Research Institute for the Environment and Livelihoods Charles Darwin University Darwin Northwest Territories Australia; ^4^ Carrera de Biología Marina Universidad Científica del Sur Lima Peru; ^5^ School of Science, Technology and Engineering University of the Sunshine Coast Hervey Bay Australia; ^6^ Marine Megafauna Foundation West Palm Beach Florida USA; ^7^ KAUST Coral Restoration Initiative (KCRI) King Abdullah University of Science and Technology Thuwal Saudi Arabia; ^8^ Department of Environmental Protection and Regeneration Red Sea Global Riyadh Saudi Arabia; ^9^ ICAR–Central Marine Fisheries Research Institute Kochi Kerala India; ^10^ Midaf Nature Conservation Society Bandar Abbas Iran; ^11^ Nuwat for Environmental Research & Education Janabiyah Bahrain; ^12^ Evolutionary Genomics Laboratory, Department of Biology New York University Abu Dhabi Abu Dhabi UAE; ^13^ Hopkins Marine Station Stanford University Pacific Grove California USA; ^14^ Miyaru—Shark Programme Fuvahmulah Maldives; ^15^ Wildlife Conservation Society—India Program Bangalore India; ^16^ Kish Island Fisheries Office Kish Island Iran; ^17^ Marine Megafauna Conservation Organisation (MMCO) Blue Water Divers Building Trou aux Biches Mauritius; ^18^ Persian Gulf and Oman Sea Ecology Research Center Iranian Fisheries Sciences Research Institute (AREEO) Bandar Abbas Iran; ^19^ Shark Citizen Association La Réunion France; ^20^ Wildlife Conservation Society—Western Indian Ocean Shark Program Bronx New York USA; ^21^ South African Institute for Aquatic Biodiversity Makhanda South Africa; ^22^ Nature Friends of Maldives Malé Maldives; ^23^ University of Plymouth Plymouth UK; ^24^ Department of Human Sciences and Sciences of Innovation for the Territory (DiSUIT) University of Insubria Como Italy; ^25^ Sea Mammal Research Unit, Scottish Oceans Institute University of St Andrews Fife UK; ^26^ Marine Mammal Protected Areas Task Force Joint Initiative of the IUCN SSC and WCPA Gland Switzerland; ^27^ Biology Department Woods Hole Oceanographic Institution Woods Hole Massachusetts USA; ^28^ Blue Safari Seychelles Mahé Seychelles; ^29^ Alphonse Foundation, Alphonse & St François Atolls Outer Islands Seychelles; ^30^ The Manta Trust Dorset UK; ^31^ SOSF‐D'arros Research Centre (SOSF‐DRC) Save Our Seas Foundation (SOSF) Geneva Switzerland; ^32^ Centre for Sustainable Tropical Fisheries and Aquaculture James Cook University Townsville Queensland Australia; ^33^ Maldives Whale Shark Research Programme Dhigurah Maldives; ^34^ School of Marine Science and Policy, College of Earth, Ocean & Environment University of Delaware Lewes Delaware USA; ^35^ University of Cape Town Cape Town South Africa; ^36^ Coastal Oregon Marine Experiment Station Oregon State University, Hatfield Marine Science Center Newport Oregon USA; ^37^ Oceanographic Institute of Mozambique Maputo Mozambique; ^38^ Wildtrust Pietermaritzburg South Africa; ^39^ School of Life Sciences University of KwaZulu‐Natal Durban South Africa; ^40^ Mascarene Archipelago Elasmobranch Observatory (MAEO) Agence de Recherche Pour la Biodiversité à La Réunion (ARBRE)—Biodiversity Research Agency of Reunion Island Saint Gilles France; ^41^ Marine Megafauna Foundation Centro de Investigação Científica Megafauna Marinha Praia do Tofo Mozambique; ^42^ Dalhousie University Department of Biology Halifax Nova Scotia Canada; ^43^ Institute of Zoology Zoological Society of London London UK; ^44^ Oceanographic Research Institute Durban KwaZulu‐Natal South Africa; ^45^ Madagascar Whale Shark Project Foundation Nosy‐Be Madagascar; ^46^ Wiss Federal Institute of Aquatic Science and Technology (Eawag) Dübendorf Switzerland; ^47^ Eidgenössische Forschungsanstalt für Wald, Schnee Und Landschaft (WSL) Birmensdorf Switzerland; ^48^ Pacific Shark Research Center, Moss Landing Marine Laboratories San Jose State University Moss Landing California USA; ^49^ Steering Committee, Species Survival Commission International Union for Conservation of Nature (IUCN SSC) Amman Jordan; ^50^ University of Bahri Khartoum Sudan; ^51^ Zoology Department, Faculty of Science Al‐Azhar University Assiut Egypt; ^52^ Sea Around Us—Indian Ocean, School of Biological Sciences University of Western Australia Crawley WA Australia; ^53^ National Institute of Water and Atmospheric Research (NIWA) Wellington New Zealand; ^54^ University of Tasmania Hobart Tasmania Australia; ^55^ Ruhr University Bochum Bochum Germany; ^56^ Deutsche Elasmobranchier Gesellschaft e.V University of Hamburg Hamburg Germany; ^57^ GIP—Centre Sécurité Requin ZA Pointe Des Châteaux Saint‐Leu La Réunion France; ^58^ ICAR Central Coastal Agricultural Research Institute Old Goa Goa India; ^59^ Interdisciplinary Centre for Conservation Science, Department of Biology University of Oxford Oxford UK; ^60^ EDGE of Existence Programme Zoological Society of London London UK; ^61^ Maldives Manta Conservation Programme (MMCP) M. Kureli. Buruzu Magu, Maafannu Male Maldives; ^62^ University of Victoria Victoria British Columbia Canada; ^63^ University of Exeter Cornwall UK; ^64^ School of Biological and Marine Sciences University of Plymouth Plymouth UK; ^65^ University of Gothenburg Göteborg Sweden; ^66^ Mission Blue Napa California USA; ^67^ ARC Centre of Excellence for Coral Reef Studies James Cook University Townsville Australia; ^68^ Coastal and Offshore Resources Research Center, Fisheries Research Institute Council of Agriculture Kaohsiung City Taiwan; ^69^ Institute of Marine Ecology and Conservation National Sun Yat‐Sen University Kaohsiung Taiwan; ^70^ Ministry of Fisheries and Blue Economy Mogadishu Somalia; ^71^ City University of Mogadishu Mogadishu Somalia; ^72^ Lancaster Environment Centre Lancaster University Lancaster UK; ^73^ Université de La Réunion UMR Entropie Saint‐Denis La Réunion France; ^74^ Department of Science & Technology Kavaratti Lakshadweep India; ^75^ Lakshadweep Atoll Research Foundation Lakshadweep India; ^76^ Department of Environmental Studies Ashoka University Sonepat Haryana India; ^77^ Department of Fisheries and Aquatic Sciences University of Eldoret Eldoret Kenya; ^78^ Scientific Services, South African National Parks Cape Research Centre Cape Town South Africa; ^79^ Seychelles Islands Foundation Victoria PO Seychelles; ^80^ Tanzania Fisheries Research Institute Dar es Salaam Tanzania; ^81^ Save Our Seas Foundation (SOSF) Geneva Switzerland; ^82^ Department of Zoology University of Cambridge Cambridge UK; ^83^ Silliman University Dumaguete City Negros Oriental Philippines; ^84^ National Museum of Somalia Mogadishu Somalia; ^85^ Centre for Marine Living Resources and Ecology (CMLRE) Atal Bhavan Kochi Kerala India; ^86^ Marine Conservation Society Seychelles Mahé PO Seychelles; ^87^ Emirates Nature—World Wide Fund for Nature Dubai UAE; ^88^ Departamento de Ecología e Hidrología Universidad de Murcia, Facultad de Biología Murcia Spain; ^89^ School of Ocean Sciences Bangor University Anglesey UK; ^90^ Marbec, Univ Montpellier, CNRS, IFREMER, IRD Sète France; ^91^ Department of Ichthyology and Fisheries Science Rhodes University PO South Africa; ^92^ Department of Agricultural & Food Science, Faculty of Agriculture University of Mauritius Réduit Moka Mauritius; ^93^ Faculty of Fisheries Sciences Hokkaido University Hakodate Hokkaido Japan; ^94^ Indian Ocean Tuna Commission (IOTC) Victoria Seychelles; ^95^ Environment Seychelles Victoria Seychelles; ^96^ Nelson Mandela University University Way Gqeberha South Africa; ^97^ Save Our Seas Foundation D'arros Research Centre (SOSF‐DRC) Amirantes Seychelles; ^98^ Fuvahmulah Dream NGO, Fuvahmulah Dive School Fuvahmulah Maldives; ^99^ School of Marine and Atmospheric Sciences Stony Brook University Southampton New York USA; ^100^ University of the Sunshine Coast Sippy Downs Queensland Australia; ^101^ School of Geography and the Environment Oxford University Centre for the Environment, University of Oxford Oxford UK; ^102^ Coastal Oceans Research and Development in the Indian Ocean (CORDIO) East Africa Mombasa Kenya; ^103^ Department of Environment of Hormozgan Province Bandar Abbas Iran; ^104^ Faculty of Biology, Medicine & Health The University of Manchester Manchester UK; ^105^ Qeshm Environmental Conservation Institute (QECI) Qeshm Island Iran; ^106^ Sundive Research Byron Bay New South Wales Australia; ^107^ Pwani University Kilifi Kenya; ^108^ Shark Research Institute Princeton New Jersey USA; ^109^ Universidade Lúrio (Lúrio University) Nampula Mozambique; ^110^ Fundação Quirimbas Pemba Mozambique; ^111^ Department of Blue Economy Ministry of Fisheries and the Blue Economy Antananarivo Madagascar; ^112^ Ministry of Environment and Sustainable Development (Ministère de Environnement et du Développement Durable) Antananarivo Madagascar; ^113^ Evolutionary Ecology Group, Department of Zoology University of Cambridge Cambridge UK; ^114^ Leibniz Centre for Tropical Marine Research (ZMT) Bremen Germany; ^115^ University of Bremen Bremen Germany; ^116^ Institute of Laboratory Medicine (ILM), Faculty of Medical Sciences Private University in the Principality of Liechtenstein (UFL) Triesen Liechtenstein; ^117^ Ocean Science and Solutions Applied Research Institute Education Research and Innovation Foundation, NEOM Tabuk Saudi Arabia; ^118^ Future Seas Global SPC Mina Al Fahal Muscat Sultanate of Oman; ^119^ James Cook University Townsville Queensland Australia

**Keywords:** biodiversity, conservation, fisheries, global biodiversity framework, marine spatial planning, threatened species

## Abstract

The Western Indian Ocean (WIO) is known for its high diversity of chondrichthyans (sharks, rays, and chimaeras). However, intense fishing pressure has led to severe population declines and local extinctions of several species. The Important Shark and Ray Area (ISRA) process is a collaborative, evidence‐based approach used to identify critical habitat for chondrichthyans. We analysed ISRAs across the WIO to quantify the diversity of research methods used to identify them, evaluate spatial overlap with designated marine protected areas (MPAs), model the influence of several species‐ and jurisdiction‐specific variables on ISRA delineation, and explore the importance of incorporating unpublished data into the delineation process. In total, 125 ISRAs (covering > 2.8 million km^2^; ~10% of total regional surface area) were identified within the WIO from surface waters to ~2000 m depth. These ISRAs contain over one‐third (*n* = 104, 39%) of the 270 chondrichthyan species reported from the region, with 76% being threatened with extinction according to the IUCN Red List of Threatened Species. The underlying evidence supporting ISRA identification was primarily drawn from relatively inexpensive research methods, such as visual census (25%) or fish‐market/landing site surveys (22.6%), as well as citizen science (9.5%). Incorporating unpublished records substantially increased the frequency of ISRA delineation, leading to expanded taxonomic and geographic coverage. Still, the full dataset was influenced by the same biases as the published record, tending to favour large‐bodied, wide‐ranging, and shallow‐dwelling species. Only 7.1% of ISRAs are within designated MPAs, with just 1.2% in fully protected no‐take areas. The highest no‐take overlap occurs in the Seychelles and Chagos Archipelago. These findings highlight the shortfalls in spatial protection of chondrichthyan habitats, but also present a strategic opportunity for policy‐makers and resource managers to improve current MPA coverage and meet their commitments under international agreements, such as the Global Biodiversity Framework.

## Graphical abstract

Delineation of Important Shark and Ray Areas (ISRAs) In the Western Indian Ocean was enhanced by the verification and incorporation of unpublished records. Even with this expanded dataset, ~99% of ISRA coverage fell outside of fully protected, no‐take MPAs.

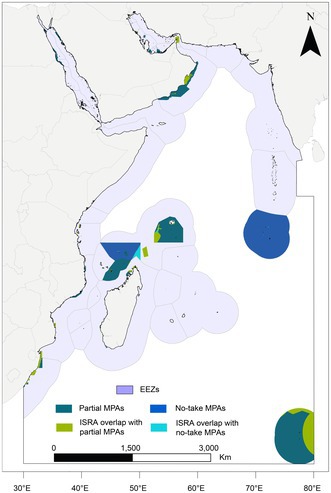

## 1 Introduction

Ongoing biodiversity loss driven by human activities has been described as the onset of a sixth global mass extinction (Cowie et al. 2022), with the recent disappearance of particularly vulnerable species potentially foreshadowing broader losses (Keck et al. 2025). Many chondrichthyans (sharks, rays, and chimaeras) are experiencing severe population declines primarily caused by overfishing, with the global abundance of the entire class reduced by half since 1970 (Dulvy et al. 2024). Chondrichthyans now represent the second‐most threatened class of vertebrates after amphibians, with over one‐third (37.5%) of species considered threatened with extinction (Dulvy et al. 2021; IUCN 2025). In the past 25 years, only three of the 1250+ chondrichthyan species assessed globally have exhibited sufficient recoveries to justify improving their global extinction risk status (Dulvy et al. 2024; IUCN 2025). However, these cases are overshadowed by that of the Java stingaree *Urolophus javanicus* , which was assessed as Extinct in March 2023 (Constance et al. 2023), marking the first recorded marine fish extinction directly linked to human activities. Further, the lost shark *Carcharhinus obsoletus* and the Red Sea *torpedo Torpedo suessii* have been assessed as Critically Endangered—Possibly Extinct (White et al. 2019; Dulvy et al. 2020; Constance et al. 2024). All three species were/are characterised by geographically restricted distributions occurring in regions subject to prolonged and intensive fishing activities, with little management and insufficient data collection (Jabado et al. 2018; IUCN 2025).

The Western Indian Ocean (WIO) accounts for ~8% of the world's ocean area, exhibits rich faunal diversity with high endemism, and provides livelihood and food for millions of people through marine fisheries (e.g., van der Elst et al. 2005; Wafar et al. 2011; Bullock et al. 2021). The region is also considered a global ‘dark spot’ for chondrichthyan conservation, whereby its high chondrichthyan diversity is coupled with elevated fishing pressure, resulting in severe population declines for many species (Jabado et al. 2018; Dulvy et al. 2024; Pollom et al. 2024; Osuka et al. 2025). Almost half (45%) of the 270 chondrichthyan species in the WIO are considered threatened with extinction, according to the IUCN Red List of Threatened Species (Jabado et al. 2018; Pollom et al. 2024; IUCN 2025), representing a higher proportion of threatened species than the most recent global estimate (Dulvy et al. 2021). This includes 28 species (10%) considered Critically Endangered, illustrating the acute risk faced by several taxa in the region. The heightened vulnerability of WIO chondrichthyans is driven largely by political and management factors: widespread artisanal and industrial fishing with limited regulation, harmful subsidies, weak enforcement capacity across many jurisdictions, and heavy reliance on sharks and rays for food and livelihoods (Jabado et al. 2018; Temple et al. 2018; Bennett et al. 2022; Pollom et al. 2024). These pressures are compounded by some of the highest estimated rates of illegal, unreported, and unregulated (IUU) fishing globally (Agnew et al. 2009). Indeed, recent analyses suggest more than a third of fishing effort in the Southwest Indian Ocean may be IUU, representing annual losses exceeding US$142 million (WWF 2023). These factors, among others, place many WIO chondrichthyan stocks under unsustainable pressure, contributing to the widespread population declines already documented in the region (Jabado et al. 2018; Dulvy et al. 2021, 2024; Pollom et al. 2024).

Developing and implementing effective management measures for chondrichthyans in this region is hindered by the diverse ecologies and conservation needs among species as well as the wide variation in socio‐economic and governance capacity across regional jurisdictions (van der Elst et al. 2005; Wafar et al. 2011; Dulvy et al. 2017; Samoilys et al. 2025). Fisheries management, research capacity, and data availability vary widely across this region (Bennett et al. 2022). To improve the regional knowledge base on chondrichthyans, scientists along with non‐academic stakeholders have employed a wide variety of research methods, including visual census (e.g., O'Connor and Cullain 2021), landing site surveys (e.g., Henderson et al. 2007), surveys of local ecological knowledge (e.g., Almojil 2021), citizen science initiatives (e.g., Wambiji et al. 2022), electronic tracking of animal movements (e.g., Daly et al. 2023), and remote video systems (e.g., Mateos‐Molina et al. 2024) to collect data within the region. Still, research effort is not evenly distributed among jurisdictions (e.g., Cochran et al. 2024) and much of the available ecological data remain unpublished and inaccessible to the wider research and conservation communities (Purgar et al. 2022).

Integrating existing data and knowledge from diverse sources is essential for ensuring a robust knowledge base to guide evidence‐based management, threat assessments, and conservation actions. Information on the function and distribution of critical chondrichthyan habitat is especially important for area‐based management strategies such as Marine Protected Areas (MPAs) (UNEP‐Nairobi Convention and WIOMSA 2021; Hyde et al. 2022). Area‐based management strategies are ideal for conserving highly resident species, but for mobile or migratory species, their effectiveness depends on capturing critical life‐history areas (including migration corridors) and ensuring connectivity across regular and predictable key habitats (Osieck et al. 1981; Knip et al. 2012; Hoyt and Notarbartolo di Sciara 2021; Goetze et al. 2024). Chondrichthyans have not usually been prioritised in MPA design and this, along with the limited available data, has contributed to a mismatch between the boundaries of MPAs and the habitats essential for key life‐history processes of these species (Hyde et al. 2022; Faure‐Beaulieu et al. 2023; Mouton et al. 2024). In the WIO, this disparity is further compounded by the fact that most current MPAs are located in shallow waters and are primarily designed to conserve coral reef ecosystems (UNEP‐Nairobi Convention and WIOMSA 2021). Although these may offer some protection for coastal or reef‐associated chondrichthyan species, such MPAs likely provide only partial or no protection for species with geographic ranges that regularly or ontogenetically extend to offshore and deepwater habitats (Dwyer et al. 2020; Samoilys et al. 2025).

Ongoing international efforts to expand global MPA coverage and enhance MPA network connectivity represent an opportunity to improve conservation potential for threatened chondrichthyans. For instance, Target 3 of the Kunming‐Montreal Global Biodiversity Framework (GBF) aims to conserve and manage at least 30% of marine habitat through protected areas and other effective area‐based conservation measures by 2030 (CBD 2022). To encourage the inclusion of high‐priority, ecologically important areas for chondrichthyan species into national marine spatial planning processes, the IUCN Species Survival Commission Shark Specialist Group (IUCN SSC SSG) launched the Important Shark and Ray Areas (ISRA) initiative (Hyde et al. 2022). The ISRA process is a collaborative, evidence‐based approach used to identify ‘discrete, three‐dimensional portions of habitat, important for one or more chondrichthyan species, that are delineated and have the potential to be managed for conservation’ (Hyde et al. 2022). ISRAs parallel other taxon‐specific approaches such as Important Bird Areas (Donald et al. 2019) and Important Marine Mammal Areas (Tetley et al. 2022), but apply this model to chondrichthyans (Hyde et al. 2022). Unlike broader frameworks such as Key Biodiversity Areas (IUCN 2016) and Ecologically or Biologically Significant Marine Areas (Clark et al. 2014), which identify sites of significance for overall biodiversity, ISRAs highlight habitats that are uniquely critical to chondrichthyan survival and life history (e.g., reproductive areas, feeding areas, migratory corridors). In this way, ISRAs complement existing biodiversity prioritisation tools by ensuring that the needs of chondrichthyans are explicitly incorporated into area‐based planning (Hyde et al. 2022; Boyd et al. 2025).

ISRA delineation employs a set of criteria developed specifically to represent chondrichthyan vulnerability, geographic ranges, life‐history, distinctiveness, and diversity (Hyde et al. 2022). Although not legally binding, ISRAs offer a powerful tool to support area‐based management and facilitate conservation actions tailored to species' needs (Mouton et al. 2024). The process of identifying ISRAs through regional collaboration can guide the development of targeted management actions, such as fisheries regulations, enforcement priorities, or the establishment of protected areas, enabling measures that are both ecologically relevant and applicable to the specific socio‐economic context of each jurisdiction (Hyde et al. 2022; Mouton et al. 2024). This process is ongoing, with delineations now complete for 9 of the 13 ISRA regions (https://sharkrayareas.org/e‐atlas/). Here, we describe and analyse ISRAs delineated within the WIO by: (1) summarising the ISRA Criteria applied by species and jurisdiction, (2) evaluating the research methods used to collect the underlying evidence, (3) assessing the relative contributions of published and unpublished information, (4) quantifying the overlap of each ISRA with spatial features such as Exclusive Economic Zones (EEZs), MPAs, and known species ranges within the WIO, and (5) examining the effects of species and jurisdictional influences on the delineation of ISRAs in the region. Our findings demonstrate the value of ISRAs both as key sites for expanding research and as actionable spatial guidance for national marine spatial planning, regional fisheries management, and global biodiversity frameworks such as the GBF.

## 2 Methods

### 2.1 Study Area

**FIGURE 1:**
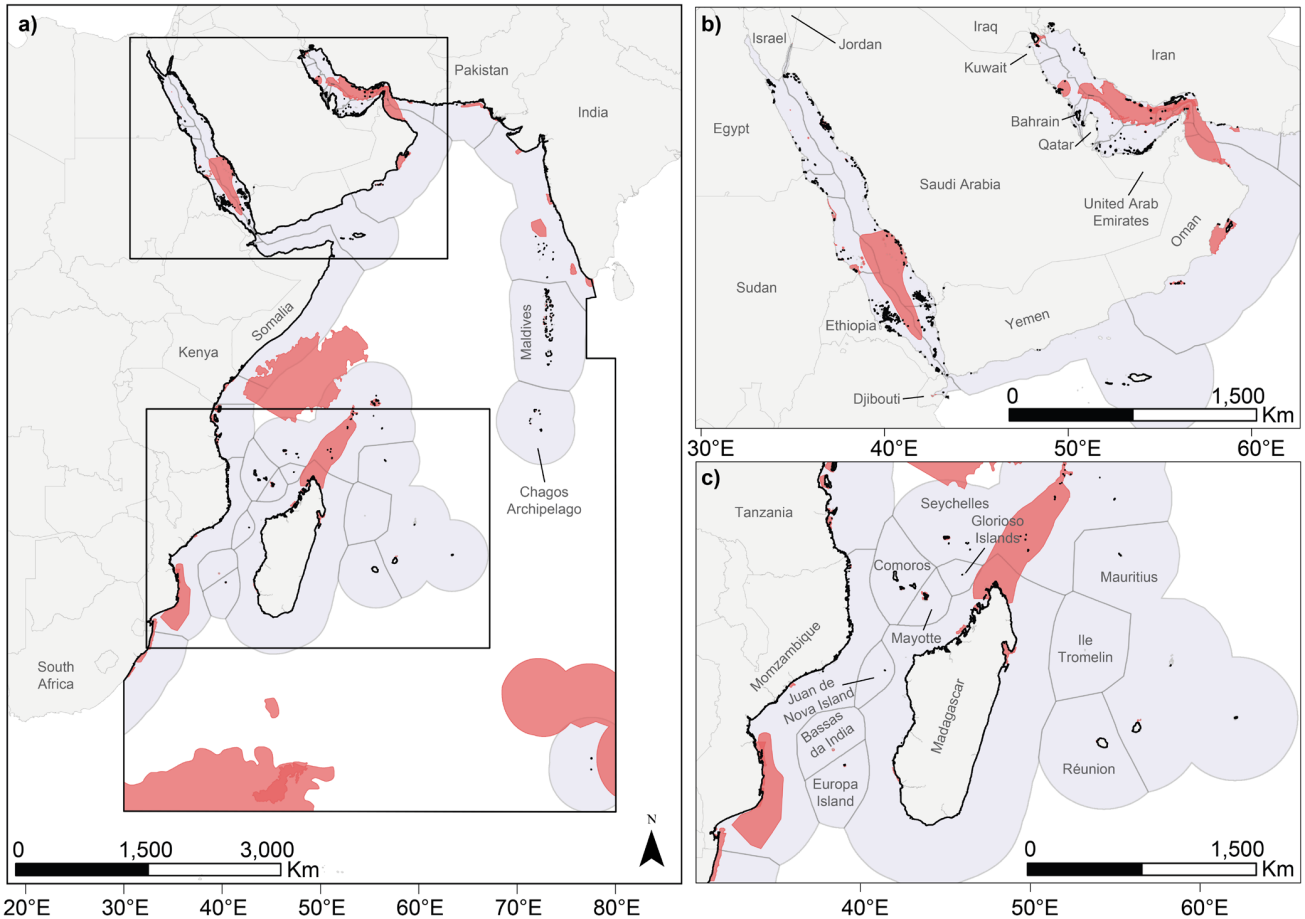
The Western Indian Ocean Important Shark and Ray Areas (ISRAs) region (black outline), with the 125 ISRAs (red), and Exclusive Economic Zones (EEZs; violet‐grey). Each country and major territory is labelled, either in the main regional map (a), or in the inset maps of the Arabian Peninsula (b) and Madagascar and the surrounding Islands (c). Maritime areas outside of the marked EEZs are considered Areas Beyond National Jurisdiction (ABNJ). Finer resolution imagery of individual ISRAs within the region is available via the interactive ISRA eAtlas available here. The boundaries and names shown and the designations used on this map do not imply the expression of any opinion whatsoever on the part of the authors concerning the legal status of any country or territory or the delimitation of its frontiers and boundaries.

The WIO (as defined through the ISRA process; IUCN SSC SSG 2024) covers more than 28 million km^2^, encompassing 29 national jurisdictions and Areas Beyond National Jurisdiction (ABNJ; Figure 1). This region extends from approximately 20° N to 35° S and from 20° E to 80° E, bounded to the west from the KwaZulu‐Natal Province (east coast of South Africa) to the northern most point of the Red Sea and extending eastward through the Arabian Sea and the Arabian/Persian Gulf to the southern tip of the Indian subcontinent and the Maldives and Chagos Archipelagos. The study area largely aligns with the Food and Agriculture Organisation of the United Nations (FAO) Fishing Area 51, with marginal expansions to fully encompass four Large Marine Ecosystems (LMEs): the Red Sea, the Arabian Sea, the Somali Current, and the Agulhas Current (Sherman and Duda 1999). These LMEs incorporate major oceanographic features that influence chondrichthyan distributions, including the Somali Current and seasonal upwelling system, the East African Coastal Current, the Mozambique Channel eddy system, and the Agulhas retroflection. Habitats across the WIO are equally diverse, ranging from inshore estuarine and riverine systems (e.g., the Rufiji delta, Tana River, and Pagani estuary), extensive coral reefs (e.g., northern Mozambique, Comoros, Seychelles, Madagascar), to large mangrove and seagrass complexes (e.g., Kenya, Tanzania, Madagascar), broad continental shelf areas (e.g., Somalia, Mozambique), and deep‐sea habitats including seamounts and submarine canyons (Jabado et al. 2023).

Chondrichthyan species across the WIO occupy a wide variety of these habitat types that can be broadly classified into coastal (*n* = 137 species, 51%) (Sherman et al. 2023), pelagic (*n* = 27 species, 10%) (Pacoureau et al. 2021), and deepwater environments > 200 m depth (*n* = 106 species, 39%) (Finucci et al. 2024). Overall, there are 270 chondrichthyan species from 54 families with confirmed reports from this region, comprising 147 sharks, 114 rays, and nine chimaeras and representing ~21% of total global chondrichthyan diversity (Weigmann et al. 2024; IUCN 2025). Almost half of these species (*n* = 123, 46%) are currently threatened with extinction, with 28 (10%) classified as Critically Endangered, 48 (18%) as Endangered, and 47 (17%) as Vulnerable. The remaining 147 species are assessed as either Near Threatened (*n* = 34, 13%), Least Concern (*n* = 68, 25%), or Data Deficient (*n* = 45, 17%) (IUCN 2025).

### 2.2 ISRA Delineation

The WIO was the third of 13 regions considered in the ISRA process, with a workshop held in Durban, South Africa, in September 2023. In total, 237 experts contributed to the ISRA process in this region, either in person or online, collating contemporary information (collected within the last 15 years) and proposing candidate ISRAs to be evaluated by an Independent Review Panel through a structured process. Each proposal was assessed against the ISRA Criteria by at least two experts, with supporting evidence assessed against minimum thresholds (as outlined in Hyde et al. 2022 and IUCN SSC SSG 2024), and final decisions on the status of a proposal were made by the Chair of the Independent Review Panel. Delineation of an ISRA depended on the successful application of one or more of the ISRA Criteria encompassing: Vulnerability (Criterion A), Range Restricted (Criterion B), Life‐History (Criterion C), and Special Attributes (Criterion D) (Hyde et al. 2022). ISRA Criteria C and D are further divided into seven sub‐criteria: Reproductive Areas (C1), Feeding Areas (C2), Resting Areas (C3), Movement Areas (C4), Undefined Aggregations (C5), Distinctiveness (D1), and Diversity (D2). Should the ISRA Criteria be successfully applied to a species, it is included as a ‘Qualifying Species’ in the respective ISRA.

Criterion A (Vulnerability) requires that at least one additional criterion be met for ISRA delineation, whereas Criterion B (Range Restricted) or Sub‐criterion D2 (Diversity) depends on the restriction or overlap of a species' geographic range, respectively (Hyde et al. 2022). To qualify under Criterion B (Range Restricted), a species needs to have a geographic range almost entirely confined to two or fewer LMEs, noting that in some cases allowances were made on a case‐by‐case basis for marginal presence in a third LME. Areas delineated under Sub‐criterion D2 needed to meet a minimum threshold of 22 Qualifying Species within an area. This region‐specific threshold was determined by calculating 30% of the maximum species richness observed across the WIO region using a 1 km × 1 km grid (IUCN SSC SSG 2024).

Species richness was estimated by overlaying the geographic ranges of individual species within the region, as defined by their IUCN Red List assessments, and summing the presence of each species within each grid cell. To calculate species richness, the geographic ranges for non‐pelagic species were refined to their known bathymetric limits according to the IUCN Red List (IUCN 2025) and Ebert et al. (2021). All overlap analyses were performed using the intersect tool on ArcGIS Pro 3.4 (Esri 2024), and the *calculate geometry* tool was used to calculate surface area in the Cylindrical Equal Area—ESRI 53034 projection. All respective ISRAs were merged into a single polygon to ensure surface area for overlapping areas was not counted twice. Each proposed area accepted by the independent review panel was documented in a factsheet available on the ISRA eAtlas (https://sharkrayareas.org/e‐atlas/). These factsheets detail the boundaries of the area, key habitat features, Qualifying Species, the respective ISRA Criteria met, and a comprehensive list of the sources used to support the delineation of the area. Full methodological details of the ISRA process are publicly available in Hyde et al. (2022) and the ISRA Guidance Document (IUCN SSC SSG 2024).

### 2.3 Classification of Research Methods

Cited research methods were extracted from the ISRA factsheets and grouped into 12 categories comprising: scientific fishing, fish‐market/landing‐site surveys, fisheries observer/logbook data, citizen science, local ecological knowledge, informal researcher observations, electronic tracking, mark‐recapture, visual census, remote video, aerial surveys, and biochemical analysis. Descriptions of each research method are available in the Supporting Information (Table S1). Each research method was counted once for each unique combination of ISRA, Qualifying Species, and Criterion (ISRA‐Species‐Criterion combinations), regardless of the number of publications or unpublished datasets referenced. Research methods used to justify multiple criteria or species within an ISRA were counted separately for each. The information used to apply the ISRA Criteria was categorised as either ‘published information’, ‘unpublished information’, or ‘mixed information’ (where both published and unpublished information were used, regardless of whether the references were skewed toward either publication type). Only peer‐reviewed journal publications and textbook chapters were considered as published research for this analysis. Internal reports, preprinted research, government documents, academic theses/dissertations, local, and other grey literature were all considered unpublished sources. Information relating to ISRA Criterion A was excluded from the analysis because it exclusively referenced the respective species' global IUCN Red List assessment.

### 2.4 Spatial Overlap Analyses

The ISRA Criterion B Range Restricted and Sub‐criterion D2 Diversity depend on set thresholds of the Qualifying Species' geographic range size or overlap as described above. Not all species or locations that hypothetically met these thresholds had sufficient information to delineate an ISRA. To be considered a Qualifying Species, a species needs to occur in the delineated area ‘regularly and/or predictably’. Therefore, based on data availability, only a subset of species with a restricted geographic range, and only a subset of locations with high species diversity, met these ISRA Criteria. To assess these spatial gaps in data availability, a series of overlap analyses compared the location of delineated ISRAs to species' geographic ranges that theoretically met the conditions to qualify under Criterion B Range Restricted. Similar analyses were used to compare the delineated ISRAs to locations that met the regional diversity threshold and could potentially qualify under Sub‐criterion D2 Diversity. Comparisons were quantified as a percentage overlap between the respective species' geographic range, potentially diverse areas, and the delineated ISRAs.

Overlap analyses were also used to compare the distribution of ISRAs with EEZs and designated MPAs. The EEZ data were taken from the World Maritime Boundaries dataset published and updated by the Flanders Marine Institute (Claus et al. 2014). Most of the spatial layers from protected areas in the Western Indian Ocean region were retrieved from the World Database on Protected Areas (WDPA) (UNEP‐WCMC and IUCN 2025) in May 2025. Each polygon was clipped to retain only the aquatic area within the WIO region, using the same landmass that was used to define the ISRA boundaries (OpenStreetMap Contributors 2024). From the WDPA dataset, areas with the following two attributes were excluded: (1) predominantly or entirely terrestrial (indicated by a zero value in the Marine field of the database); and (2) international designations, such as World Heritage Sites (UNEP‐WCMC 2019; Grorud‐Colvert et al. 2021). India does not report MPAs to the WDPA. Spatial data for India were sourced directly through contributors to the ISRA process, and the information gathered was cross‐referenced with the list of MPAs included on the Ministry of Environment and Forests government website (https://wiienvis.nic.in/database/mpa_8098.aspx). An updated list of no take status for protected areas in Mauritius was also provided by contributors.

To classify MPAs based on their level of protection, they were categorised into two groups: no‐take MPAs, where all extractive activities are prohibited (aligned with IUCN categories I, II, or III, as per Day et al. 2019), and partial MPAs (also known as multiple‐use MPAs), which allow some extractive activities and align with IUCN categories IV, V, VI, or other classification types. The IUCN categories for each MPA are reported in the WDPA. MPAs that do not report their IUCN categories to WDPA were classified as partial MPAs. Overlapping polygons were merged to avoid double‐counting surface area. Surface area was recalculated for all spatial layers under the World Cylindrical Equal Area projection. The percentage area and number of ISRAs overlapping with MPAs at two designation levels (partial and no‐take MPAs) were measured. To evaluate the coverage of MPAs in each jurisdiction and determine the expansion needed to meet Target 3 of the Kunming‐Montreal GBF (CBD 2022), we analysed the proportion of EEZ, as determined by the UN Convention on Law of the Sea (UNCLOS), covered by both no‐take and partial MPAs.

### 2.5 Factors Influencing ISRA Delineation

The taxonomic and geographic distribution of published research is known to be influenced by several species‐specific and socio‐economic factors (Ducatez 2019). To evaluate the relationships between factors of interest and the probability and frequency of ISRA delineation for species and jurisdictions, including to what degree these relationships change in response to the incorporation of unpublished data into the ISRA process, a hurdle modelling approach was employed (Cragg 1971; Welsh et al. 1996). Species models explored maximum body size (in cm), geographic range area within the WIO (in km^2^), and median depth (in m) as predictor variables. Body size data were measured using taxa‐specific conventions: total length (TL) for most sharks and rays (Last et al. 2016; Ebert et al. 2021), disc width (DW) for Myliobatiformes (Last et al. 2016), and body length (BDL) for chimaeras (Compagno et al. 1990). All species data were extracted from global IUCN Red List assessments (IUCN 2025). The jurisdiction model used EEZ area (Claus et al. 2014), national chondrichthyan species richness (quantified as the number of IUCN species distributions overlapping with the portion of each jurisdiction's EEZ within the WIO region), and gross domestic product (GDP; World Bank Data 2024).

At the first stage (i.e., the hurdle component), models assessed the probability of delineating at least one ISRA for each of the 270 chondrichthyan species or 29 jurisdictions within the WIO. Subsequently, and conditional on the existence of at least one ISRA, they then modelled the total number of ISRAs delineated (i.e., the count component). The hurdle component of the model used the binomial distribution, and the count component of the model assumed a truncated negative binomial distribution in the number of ISRAs. Models were formulated as only the interaction of the variables of interest and the level of unpublished data incorporated i.e., (1) using only published sources, (2) using published and mixed sources, and (3) using the full dataset (published, unpublished, and mixed sources). Main effects were not modelled. Models were run separately for each variable of interest because the low number of replicates available for jurisdiction meant that combined analyses led to model overparameterization. This approach was maintained for the species models to ensure consistency. We conducted post hoc comparisons to identify any significant changes in the relationships between species‐specific and/or socio‐economic factors and the probability and frequency of ISRA delineation associated with the incorporation of unpublished data into the ISRA process. Integration of jurisdiction and species as random effects was considered but in both cases within‐group variation was small, particularly in the hurdle component, leading to unstable parameter estimates and/or models failing to converge. The Holm‐Bonferroni correction (Holm 1979) was applied to adjust for multiple comparisons and the resultant elevated risk of false positives. Modelling was performed in R v4.1.0 (R Core Team 2021) using *glmmTMB* and *emmeans*, model residual distributions were visually inspected using *DHARMa*.

## 3 Results

### 3.1 ISRA Delineation

**FIGURE 2:**
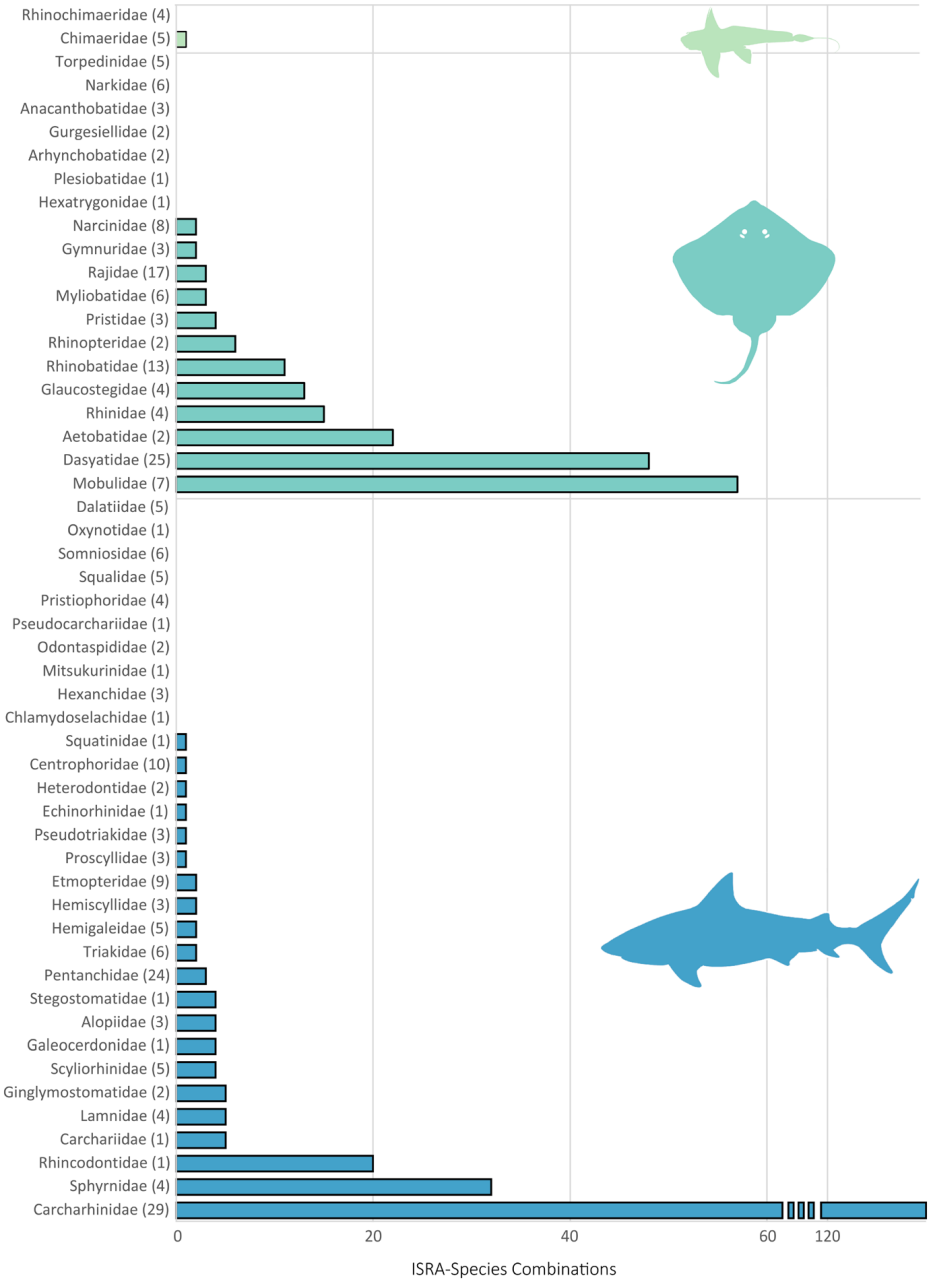
The distribution of ISRA‐Species combinations among chondrichthyan families found in the Western Indian Ocean. Numbers in parentheses next to each family name denote the number of species within that family that occur in the region. Chimaeras are shown in light green, rays in teal, and sharks in blue.

Of the 135 proposals reviewed by the Independent Review Panel, 125 had sufficient information available to be formally delineated as ISRAs within the WIO region (Jabado et al. 2023), covering more than 2.8 million km^2^ (~10%) of the WIO region's total surface area (Figure 1). The finalised ISRAs ranged in size from < 1 km^2^ to nearly 724,577 km^2^ (mean ± SD = 33,683 ± 185,604 km^2^) and were delineated from surface waters to nearly 2000 m depth (mean = 242 ± 400 m). The ISRAs included 104 Qualifying Species, representing 38.7% of all species reported from the region. Most ISRAs (*n* = 76, 61%) were delineated for multiple species (mean = 3.3 ± 4.3 species), and most Qualifying Species (58%) were included in more than one ISRA (mean = 4 ± 5.8 ISRAs), resulting in 417 unique combinations of ISRAs and Qualifying Species (hereafter: ISRA‐Species combinations) (Figure 2).

The 104 Qualifying Species comprised 52 sharks (35% of the regional diversity), 51 rays (45%), and one chimaera (11%). Examining the broad habitat classifications among species showed that pelagic species were well represented, with 20 Qualifying Species (74% of the regional total) and 131 ISRA‐Species combinations. Roughly half (52%) of the coastal species known from the region were included as Qualifying Species, with 271 ISRA‐Species combinations. Only 13 (12%) of the 106 deepwater species known from the WIO had sufficient contemporary information to delineate an ISRA, resulting in 15 unique ISRA‐Species combinations. The majority of Qualifying Species (*n* = 79, 76%) were considered threatened with extinction according to the IUCN Red List and qualified under Criterion A Vulnerability. Twenty‐eight species (27% of Qualifying Species) were considered range‐restricted, qualifying under Criterion B. Most ISRAs (*n* = 123, 98%) were delineated under Criterion C for their importance to the critical life‐history processes of 78 Qualifying Species. Twelve areas were delineated for Special Attributes (Criterion D) of 49 Qualifying Species.

### 3.2 Classification of Research Methods

**FIGURE 3:**
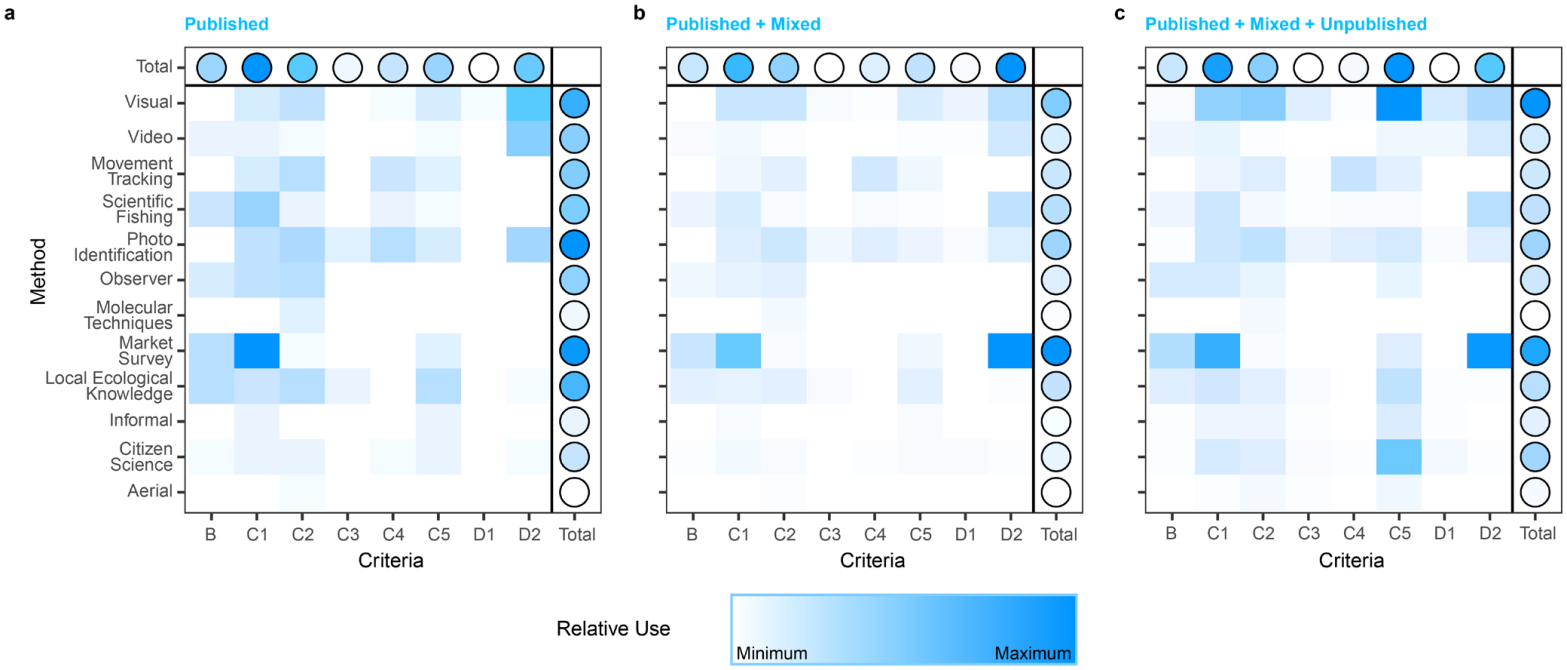
Relative use of each research method per ISRA Criteria (B—Range Restricted; C1—Reproductive Areas; C2—Feeding Areas; C3—Resting Areas; C4—Movement Areas; C5—Undefined Aggregations; D1—Distinctiveness; D2—Diversity) for (A) published data, (B) published and mixed data, and (C) the total dataset combining the published, mixed, and unpublished data.

The 12 research methods considered here (Table S1) were applied a total of 736 times across the unique ISRA‐Species‐Criterion combinations (*n* = 861, *n* = 480 after excluding Criterion A from the analysis). The total frequency of each research method applied across the entire WIO ranged between 3 and 184 instances (mean = 61.3 ± 54.9). The most frequently used research methods included visual census (*n* = 184), fish‐market/landing‐site surveys (*n* = 167), mark‐recapture (*n* = 71), and citizen science (*n* = 70) (Figure 3). The least frequently used research methods were informal researcher observations (*n* = 23), aerial surveys (*n* = 9), and biochemical analyses (*n* = 3). Overall, the four most common research methods were prevalent for all ISRA Criteria (ranging from 38% of references for Movement Areas [Sub‐criterion C4] to 76% for Diversity [Sub‐criterion D2]). Visual census was the most common method for delineating Feeding Areas (Sub‐criterion C2, 33%), Resting Areas (Sub‐criterion C3, 39%), Undefined Aggregations (Sub‐criterion C5, 72%), and Distinctiveness (Sub‐criterion D1, 52%). Fish‐market/landing‐site surveys were the most frequently used method for supporting Range Restricted (Criterion B, 38%), Reproductive Areas (Sub‐criterion C1, 36%), and Diversity (Sub‐criterion D2, 51%). Movement Areas (Sub‐criterion C4) were most frequently delineated using electronic tracking (*n* = 16, 55%), a method that supported the delineation of few ISRAs under other ISRA Criteria.

The 736 applications of research methods were further categorised as published (*n* = 226, 31%), unpublished (*n* = 347, 47%), or mixed (*n* = 163, 22%). When examining the distribution of the different data types among the 125 ISRAs, 20 ISRAs (16%) were based exclusively on published data compared to 56 ISRAs (45%) that relied exclusively on unpublished data, and 49 ISRAs (39%) were delineated using mixed information (Figure 3). Most research methods were skewed toward one of the publication categories, and infrequently used methods, such as biochemical analyses (100% published) or aerial surveys (89% unpublished), exhibited the highest skewness. However, some of the most common methods also showed clear tendencies toward one type of data over the others, including visual census (65% unpublished), fish‐market/landing‐site surveys (54% mixed), or citizen science (83% unpublished).

### 3.3 Spatial Overlap Analyses

Of the 270 chondrichthyan species assessed in the WIO, 117 (43%) had sufficiently confined distributions to potentially qualify as range‐restricted. However, only 28 (23%) of these species had adequate contemporary information to apply Criterion B. Among these, 22 species (79%) were delineated within single ISRAs, often encompassing substantial portions of their respective global ranges (mean = 16.6 ± 25.3%), while the remaining six were associated with multiple ISRAs (mean = 15.9 ± 26.1% coverage of global ranges) (see SI Figure 1). These findings underscore both the scarcity of actionable data for most range‐restricted species in the WIO and the reliance of those with data on delineated ISRAs. For example, the entire known (but poorly documented) range of the eastern dwarf false catshark *Planonasus indicus* and over half of the better‐defined range of the flapnose houndshark *Scylliogaleus quecketti* were contained within ISRAs.

Similar spatial analysis identified 1,118,331 km^2^ (4% of the WIO) as potentially meeting the species richness threshold (≥ 22 Qualifying Species) for Sub‐criterion D2 (Diversity). However, only four ISRAs covering 33,300 km^2^ (3% of potential area, 0.001% of regional waters) were delineated under this Sub‐criterion. Still, these areas encompassed 23–24 species each, including 48 unique species and 94 ISRA‐Species combinations between them. Although another 217,703 km^2^ of potentially diverse habitat was delineated under other Criteria (bringing the total coverage up to 251,003 km^2^ or 22% of potential Diversity areas), the contrast between theoretical richness and confirmed Diversity ISRAs illustrates both the high thresholds needed to apply this Sub‐criterion and the current data limitations in the WIO.

The World Maritime Boundaries dataset contained 39 EEZs for the WIO, including 31 that were undisputed and administered by one of the region's 29 national jurisdictions. The remaining eight were associated with disputed territories. Collectively, regional EEZs encompass more than 12 million km^2^, or approximately 43% of the region's surface area, with the remaining 57% representing ABNJ. Analysis of the overlap between the EEZs and delineated ISRAs showed that most of the overall ISRA area (1,917,700 km^2^, 67.59%) was located in ABNJ. However, the majority of individual ISRAs at least partially overlapped with one or more national jurisdictions (*n* = 122 ISRAs, 98%), though the number of ISRAs within each jurisdiction varied from none in Jordan to 27 in the Maldives (mean = 4.62 ± 4.94 ISRAs per country). The overlap analysis also identified eight multi‐jurisdictional ISRAs (with 2–6 associated jurisdictions per ISRA) and seven in areas with overlapping jurisdictional claims. Accounting for all of these factors yielded a final total of 143 unique pairings of ISRA and jurisdiction (hereafter: ISRA‐Jurisdiction combinations).

**FIGURE 4:**
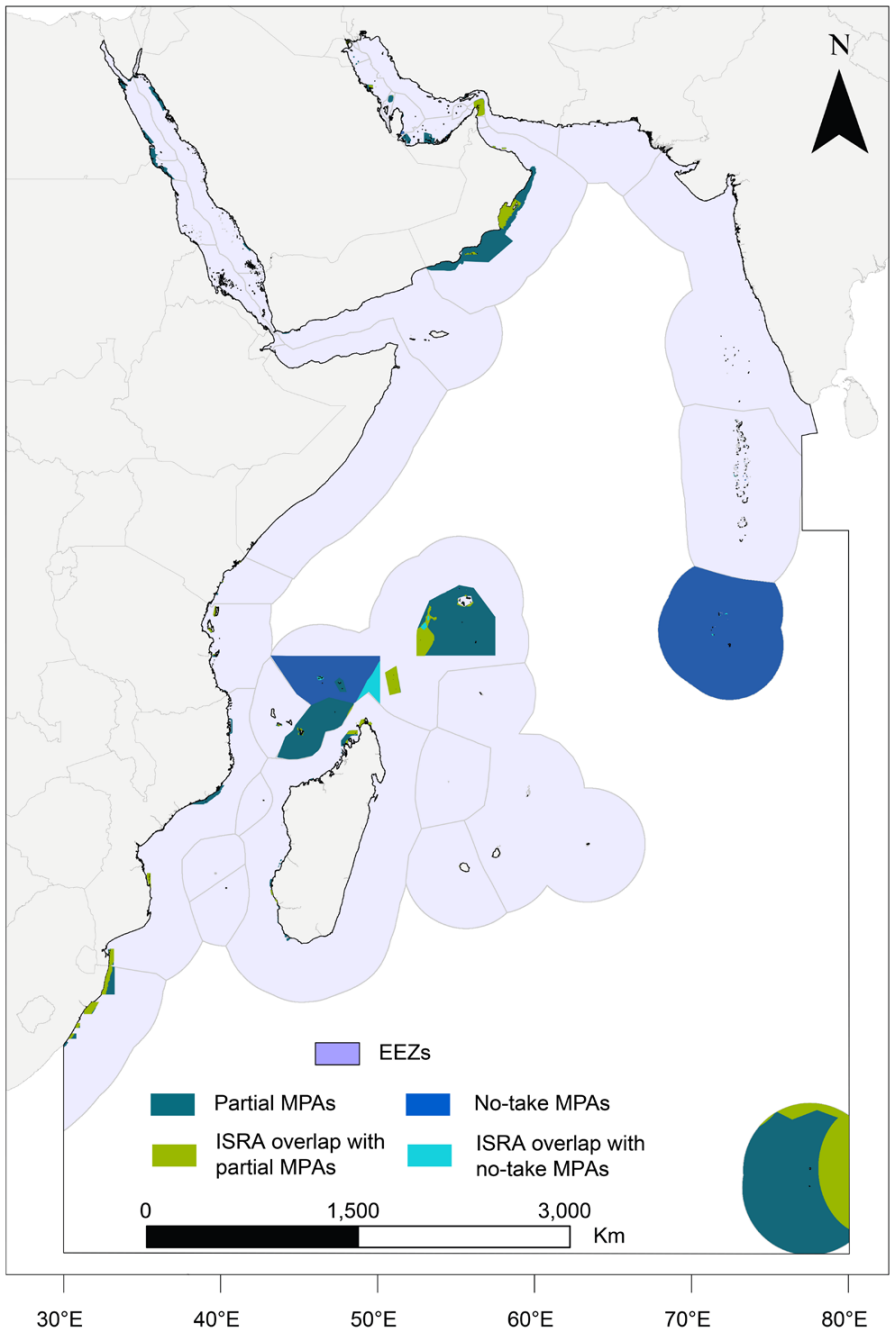
Map of the Western Indian Ocean Important Shark and Ray Areas (ISRAs) region (black outline), with the Exclusive Economic Zones (EEZs; violet‐grey). Partial MPAs and no‐take MPAs are shown in dark green and dark blue respectively. Overlap between Important Shark and Ray Areas (ISRAs) with partial MPAs is shown in light green while ISRA overlap with no‐take MPAs is shown in light blue.

**FIGURE 5:**
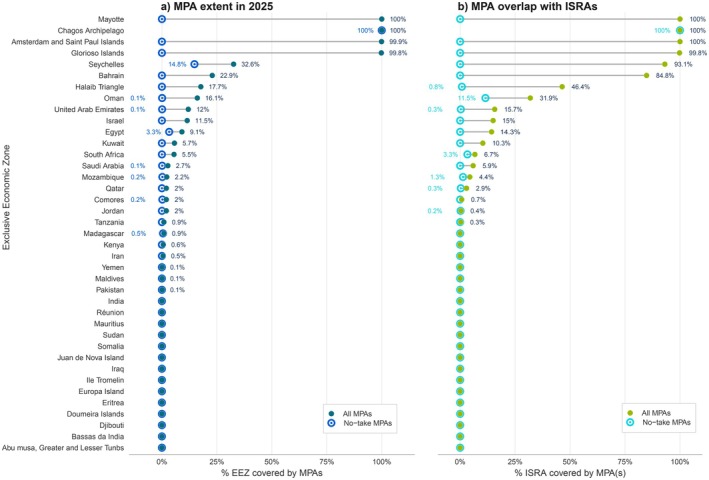
Abacus plots showing (a) the extent of MPA and no‐take MPA coverage as a percentage of Exclusive Economic Zone (EEZ) and (b) the percentage of ISRAs within each EEZ that overlap with MPAs in general and no‐take maps in specific.

The 366 protected areas in the WIO encompass nearly 1.8 million km^2^ (6.4%) of the regional ocean surface area. Over two‐thirds (69%) of the protected areas permit some level of fishing activity and are classified as partial MPAs (*n* = 253, ~940,000 km^2^), while 31% are designated as no‐take (*n* = 113; ~850,000 km^2^) (Figure 4). The overlap between existing MPAs (*n* = 121) and delineated ISRAs (*n* = 65) was limited to just 7.1% of the overall ISRA area and 11.3% of the overall MPA area. Only 1.2% (22,201.5 km^2^) of ISRAs overlapped with no‐take MPAs. Most of this overlap was within the Seychelles (98.1%, 21,814 km^2^), followed by the Chagos Archipelago (1.3%, 295.1 km^2^) (Figure 5). The remaining overlap between ISRA and no‐take MPAs was across 10 jurisdictions. There were 180,453 km^2^ of overlap between ISRAs and partial MPAs (10.1% of the total MPA coverage) in 19 jurisdictions. Of this, most of the overlap of ISRA and partial MPAs (88.3%, 159,336 km^2^) was within Amsterdam and Saint Paul Islands (56.9%, 102,588 km^2^), Seychelles (21.6%, 38,889 km^2^), and Oman (9.9%, 17,859 km^2^). As there are no designated MPAs in ABNJ, no overlap occurred with the 102,400 km^2^ of ISRAs delineated there.

### 3.4 Factors Influencing ISRA Delineation

**FIGURE 6:**
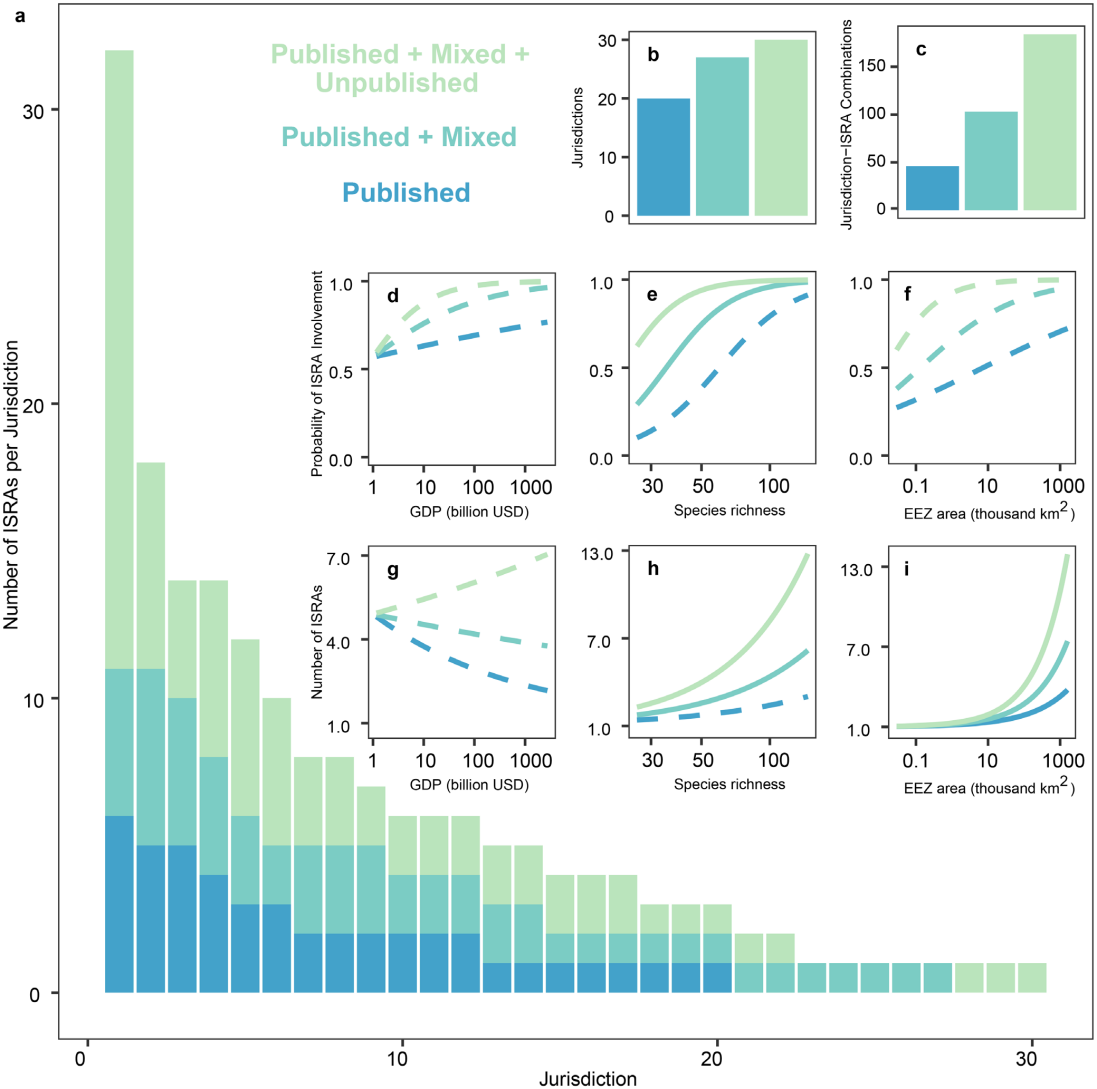
Change in the number of ISRAs per species when incorporating different data sources, the overall number of species with at least one ISRA designation, and the number of ISRA‐Species combinations with the inclusion of unpublished data (a–c). Hurdle model outputs showing changes in the probability of species being designated at least one ISRA in relation to (d) median depth, (e) maximum body size, and (f) species geographic range. Hurdle model outputs showing changes in the number of ISRAs per species in relation to (g) median depth, (h) maximum body size, and (i) species geographic range.

The use of mixed and unpublished information substantially improved the potential to apply the ISRA Criteria, resulting in higher numbers of species and ISRA‐Species combinations (Figure 6a–c). Only 50% of Qualifying Species (52 of 104) were supported by published information alone (Figure 6b). An additional 41 species were included with the addition of supporting unpublished information, and a further 11 relied entirely on unpublished records. Overall, 79% of the 417 ISRA‐Species combinations were supported by at least some unpublished information, and 40% were based exclusively on unpublished sources (Figure 6c).

The species hurdle modelling showed a higher likelihood to meet the ISRA Criteria at least once for shallow‐dwelling (*p* < 0.001, *R* ^2^ = 0.105), large‐bodied (*p* < 0.001, *R* ^2^ = 0.183), and wide‐ranging (*p* < 0.001, *R* ^2^ = 0.207) species (Figure 6d–f). Large‐bodied (*p* < 0.001, *R* ^2^ = 0.155) and wide‐ranging (*p* < 0.001, *R* ^2^ = 0.050) species were also more likely to have a higher number of designated ISRAs (Figure 6g–i). For instance, reef‐associated species such as the grey reef shark *Carcharhinus amblyrhynchos* (23 ISRAs) and spotted eagle ray *Aetobatus ocellatus* (20 ISRAs); the largest species such as the whale shark ( *Rhincodon typus* ) (20 associated ISRAs) and reef manta ray *Mobula alfredi* (34 ISRAs); and widely distributed aggregating species such as the scalloped hammerhead *Sphyrna lewini* (30 ISRAs), were among the most represented taxa. Range‐restricted species were less likely to meet the ISRA Criteria and were typically included in fewer ISRAs. However, in contrast to the trend seen for meeting the ISRA Criteria at least once, the total number of ISRAs per species decreased with shallower depth distributions (*p* < 0.001, *R* ^2^ = 0.065). This inversion of the relationship was driven primarily by deep‐diving but epipelagic species such as the whale shark (median depth of 964 m, 20 associated ISRAs), oceanic manta ray *Mobula birostris* (623 m, 7 ISRAs), and scalloped hammerhead (522 m, 30 ISRAs). In all cases, the incorporation of unpublished data led to a significant increase in the likelihood of a species being designated in at least one ISRA (*p* < 0.002) and in the total number of ISRAs designated for those with at least one ISRA (*p* < 0.002). Full model outputs for species analyses are available in the Supporting Information (Tables S2 and S3).

**FIGURE 7:**
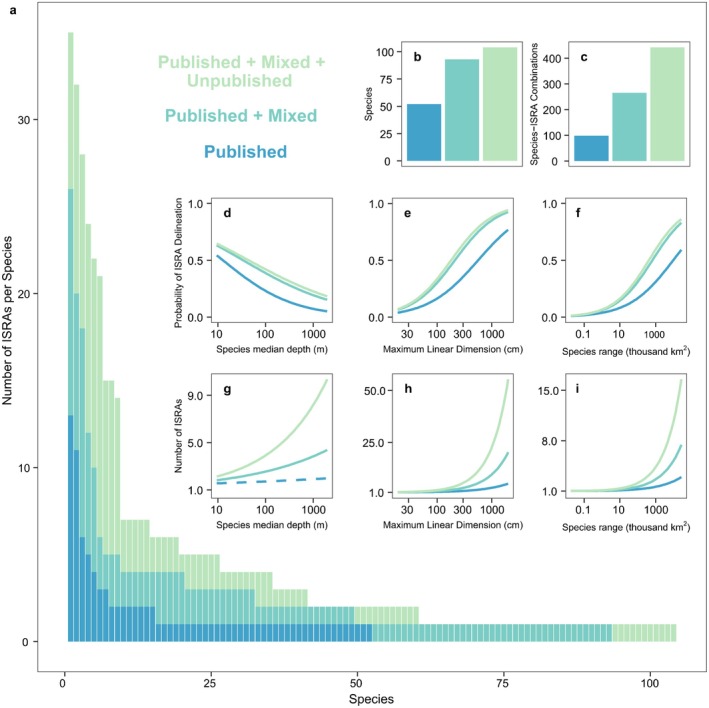
Change in the number of ISRAs per jurisdiction, the overall number of jurisdictions with at least one ISRA designation, and the number ofISRA‐Jurisdiction combinations with the inclusion of unpublished data (a–c). Hurdle model outputs showing changes in the probability of jurisdictions being designated at least one ISRA in relation to (d) gross domestic product (GDP), (e) species richness, and (f) Economic Exclusive Zone (EEZ) area. Hurdle model outputs showing changes in the count of ISRA designations by jurisdiction in relation to (g) GDP, (h) species richness, and (i) EEZ area.

Incorporating mixed and unpublished information also increased the number of jurisdictions in which ISRAs were delineated when compared to the published record alone (Figure 7a–c). Of the 29 national jurisdictions within the WIO, 18 hosted at least one ISRA that was entirely supported by published research, while seven were included on the basis of mixed records, and three were supported exclusively by unpublished information (Figure 7b). The frequency of ISRA delineation among jurisdictions was also substantially increased, with only 28 (20%) of the 143 ISRA–Jurisdiction combinations based exclusively on published research, while 51 (36%) were based on unpublished records and 64 (44%) were based on mixed sources (Figure 7c).

The jurisdiction‐level hurdle models showed no significant trend between the probability of ISRA delineation (*p* = 0.245), total ISRA numbers (*p* = 0.153), and the GDP of host jurisdictions (Figure 7d,g). The probability of jurisdiction inclusion was always greater than 50%, meaning that even the most resource‐limited jurisdictions were more likely to host at least one ISRA than not. Similarly, EEZ size was not a significant predictor of ISRA delineation (*p* = 0.153), with most national jurisdictions hosting at least one ISRA despite wide differences in total EEZ area (Figure 7e). However, EEZ size was a significant predictor of the total number of ISRA designations (*p* < 0.001, *R* ^2^ = 0.255) (Figure 7h). Differences in chondrichthyan species richness among jurisdictions showed a significant relationship with ISRA delineation (*p* = 0.02, *R* ^2^ = 0.263) and the total number of ISRA designations (*p* = 0.016, *R* ^2^ = 0.218) (Figure 7f,i). Jordan was the only country within the region with an EEZ that did not overlap with at least one ISRA. Six other jurisdictions with limited EEZs (< 50,000 km^2^) contained just one or two ISRAs. The remaining 22 jurisdictions had a mean of 5.9 ISRAs (SD ±5.4), though there were a few outliers with large EEZs and few ISRAs (Figure 1). The archipelago jurisdictions of the Maldives and the Seychelles are among the largest EEZs in the region (0.92 and 1.34 million km^2^, respectively) and contain the most ISRAs (27 and 11, respectively). Incorporation of unpublished data led to a significant increase in the total number of ISRAs designated for those with at least one ISRA when examining trends with species richness and EEZ area (*p* = 0.002) but not in any other instance (*p* ≧ 0.123). Full model outputs for the jurisdiction analyses are available in the Supporting Information (Tables S4 and S5).

## 4 Discussion

### 4.1 ISRA Delineation, Criteria, and Qualifying Species

Delineating ISRAs in the WIO is crucial to informing and improving regional management for chondrichthyans. The ISRA process identified critical habitats for nearly 40% of the region's chondrichthyan species. However, Qualifying Species were disproportionately distributed across the ISRA Criteria. Approximately 78% of Qualifying Species met Criterion A (Vulnerability), reflecting the high proportion of threatened species in the WIO (Jabado et al. 2018; Pollom et al. 2024) and a regional tendency toward research focused on threatened species (Ducatez 2019). For example, the Rhinopristiformes are among the most imperilled vertebrate orders (Moore 2017; Jabado 2018; Kyne et al. 2020, 2024), with 54% of all species considered Critically Endangered. Recent research has addressed data gaps for this order (e.g., Elhassan 2018; Sreekanth et al. 2020; Jabado et al. 2021; Boldrocchi et al. 2023; Mateos‐Molina et al. 2024), contributing to 20 ISRA‐Species combinations. In contrast, despite a high proportion of range‐restricted species in this region (117 of 270 species), only 24% had sufficient data to support the delineation of an ISRA (28 species, 36 ISRA‐Species combinations), highlighting an underrepresentation of these species in the available data. This underrepresentation is of particular conservation concern, given known relationships between endemism and elevated extinction risk (Işik 2011; Dulvy et al. 2021), and reflected in possible extinctions of range‐restricted species in the region (e.g., Red Sea torpedo; Constance et al. 2024). Many range‐restricted species are known only from their holotype, have few occurrence records, occur in deepwater and/or ABNJ where little research has been undertaken, or have limited commercial value and are of low research priority. Research is urgently needed for range‐restricted species to ensure improved understanding of the conservation needs crucial to their long‐term survival.

The majority of ISRAs (98%) were delineated under Criterion C (Life‐History), highlighting a diversity of critical chondrichthyan habitats. Reproductive Areas (Sub‐criterion C1) were the most frequently delineated, occurring within 72 ISRAs for 58 Qualifying Species. These were primarily supported by observations of important life stages (e.g., neonates or gravid females) collected using either in situ monitoring (e.g., visual census, citizen science) or specimen collection (e.g., fish‐market/landing‐site surveys, fisheries observers, scientific fishing). Comparatively fewer Feeding Areas (C2) were represented in ISRAs (36 for 16 Qualifying Species). These were predominantly associated with predictable plankton blooms or predictable food pulses from fish spawning aggregations (e.g., Al Shaheen ISRA in Qatar), mass fish migrations (e.g., Greater Protea Banks ISRA in South Africa), and sea turtle rookeries (e.g., Southern Mwali ISRA in Comoros) (Jabado et al. 2023). Resting Areas (C3) and Movement Areas (C4) were represented by even fewer ISRAs, which may reflect the difficulty in observing and documenting resting behaviour (often based on visual census observations of benthic resting species) and the limited application of electronic tracking in the region. Tracking studies can be cost‐prohibitive and have generally been restricted to large‐bodied and often charismatic species such as whale sharks, white sharks *(Carcharodon carcharias)*, or devil rays (e.g., Berumen et al. 2014; Robinson et al. 2016; Ducatez 2019; Harris et al. 2021; Kock et al. 2022). Despite these limitations, the continued monitoring of movement patterns may be critical for the conservation of highly mobile species, especially in the context of ongoing illegal fishing pressure, even within designated MPAs (Carr et al. 2013; Jacoby et al. 2020; Harris and Stevens 2024), and potential migratory shifts in response to climate change (Hammerschlag et al. 2022; Womersley et al. 2024). Finally, Undefined Aggregations (C5) were delineated relatively frequently in comparison (70 ISRAs for 37 Qualifying Species) to the other Life‐History sub‐criteria, particularly for species and locations where wildlife tourism facilitates visual census efforts and leads to an abundance of citizen science records. For instance, Undefined Aggregations comprised 50% (*n* = 15) of ISRAs delineated for the scalloped hammerhead, and most of these (*n* = 12, 80%) were supported by unpublished visual census or citizen science data.

Few ISRAs were delineated under Criterion D (Special Attributes), which was designed to capture unique biological, behavioural, or ecological characteristics (i.e., Sub‐criterion D1—Distinctiveness) and areas of high species diversity (Sub‐criterion D2) (Hyde et al. 2022). Delineation under these sub‐criteria requires either substantial in situ observation of animal behaviour (for Distinctiveness) or evidence of the regular and predictable co‐occurrence of at least 22 species (for Diversity). The eight ISRAs delineated under Distinctiveness were predominantly cleaning stations (O'Shea et al. 2010) in the Maldives, Mozambique, or South Africa and monitored by either visual census or remote video surveys. Capturing information on such sites may be particularly important when the species do not meet other ISRA Criteria. For example, the Southern Inhambane Province ISRA in Mozambique is the only known location where smalleye stingrays *Megatrygon microps* regularly visit cleaning stations (Boggio‐Pasqua et al. 2019; Buschmann et al. 2024). The four Diversity ISRAs delineated in the region (i.e., Wadge Bank and Manjapparai in India; Unguja in Tanzania; and Southern Inhambane Province in Mozambique) have potential conservation value as key areas where management measures could benefit multiple species (23–24 species at each site), especially since 94% (*n* = 49) of the 52 unique Qualifying Species at these ISRAs are also threatened with extinction.

Reviewing, validating, and incorporating unpublished data nearly doubled the number of sources considered during the ISRA process. This corresponded to similar increases in total ISRAs delineated, diversity of Qualifying Species, and distribution among jurisdictions. These results highlight the value of citizen science (Crochelet et al. 2025), local ecological knowledge (Karnad et al. 2024), and government initiatives (Sattar et al. 2014) as complementary to the efforts of more traditional research approaches, providing key sources of additional data for policy makers and resource managers. Indeed, the resulting increased spatial and taxonomic ISRA coverage can help guide the designation of additional MPAs or the implementation of other management measures (Hyde et al. 2022). Further, the collaborative nature of the ISRA process helped to address the time lag between data collection and publication, particularly for long‐term datasets. Cooperation among researchers, stakeholders, and policy makers during MPA designation and other management processes relevant to chondrichthyans could yield similar positive results.

Identifying novel study sites could lead to increased research in a historically data‐poor region (Dulvy et al. 2024). Ongoing monitoring and research within ISRAs will be key to ensuring these critical habitats are not impacted by anthropogenic activities (Mouton et al. 2024) and that data continue to be available to ensure they meet the ISRA Criteria over time. Periodic reassessment is central to the ISRA process, ‘future‐proofing’ sites by allowing existing areas to be re‐evaluated and new ones delineated as species ranges shift in response to climate change or other ecological disruptions (Hyde et al. 2022). Although the ISRA process itself does not incorporate climate projections, managers and policymakers could use climate velocity analyses alongside ISRA designations when prioritising sites for protection, favouring areas where oceanographic conditions are projected to remain relatively stable and more likely to continue supporting chondrichthyan populations over coming decades. Research should also be expanded at the 45 sites within the WIO delineated as Areas of Interest (e.g., Dahlak Archipelago in Eritrea, Socotra Archipelago in Yemen) where the available data indicated chondrichthyan presence related to the ISRA Criteria but were insufficient to show regular and predictable use of an area (Jabado et al. 2023).

### 4.2 Diversity of Qualifying Species

Our findings demonstrate relationships between species qualification and ISRA frequency, with several functional traits that vary among taxa. Large‐bodied, wide‐ranging, and shallow‐dwelling species were best represented in the ISRAs. To some extent, this reflects conservation and resource needs, with coastal‐ and reef‐associated species being among the most threatened, and large‐bodied coastal and pelagic species being of the highest commercial and consumptive value (Sherman et al. 2023; Temple et al. 2024). Indeed, shallow‐dwelling, coastal species are the most exposed to anthropogenic threats, especially overfishing and habitat degradation (Dulvy et al. 2021), while many large‐bodied and wide‐ranging pelagic species have suffered severe population declines as a result of targeted and incidental catches in oceanic fisheries (Queiroz et al. 2019; Pacoureau et al. 2021). Still, the tendency for research to disproportionately focus on charismatic species continues to bias scientific attention across a wide range of taxa, including chondrichthyans (Ducatez 2019). These disparities in data distribution were evident in the WIO ISRA delineations, where three planktivorous megafauna species (reef manta ray, oceanic manta ray, and whale shark) accounted for 60 (14.3%) of the 417 ISRA‐Species combinations despite representing < 3% of the Qualifying Species. These species are all large‐bodied (Last et al. 2016; Ebert et al. 2021), wide‐ranging (IUCN 2025), threatened (IUCN 2025), relatively easy to identify, and regularly found in shallow coastal waters at predictable aggregation sites (Norman et al. 2017; Palacios et al. 2023), thereby improving their detectability and enabling greater research accessibility. They also possess several additional characteristics that are conducive to research, including surface‐associated feeding behaviours that are easily detectable by boat‐based visual census (Robinson et al. 2016), unique individual markings suitable for photo‐identification (Pierce et al. 2018), dedicated wildlife tourism operations to facilitate citizen science (Cisneros‐Montemayor et al. 2013), and docile temperaments that simplify the deployment of electronic tracking devices (Berumen et al. 2014). These traits make them ideal study animals, generating large volumes of research and citizen science data. The ISRA process itself can be effort‐intensive, and the number of NGOs, researchers, and citizen scientists dedicated to these charismatic species likely increased the number of submissions for mantas and whale sharks, resulting in more delineated ISRAs. Still, similar methods (e.g., photo‐identification from markings) could be expanded to other species and incorporated into visual census surveys, citizen science, and other research methods to improve data availability on understudied species.

The ISRA process highlighted several data gaps that continue to hinder conservation of several particularly susceptible taxa. Small‐bodied, range‐restricted, and deepwater species were all underrepresented in the WIO ISRAs. For example, the electric rays (Torpediniformes) are poorly studied (37% of species occurring in the WIO are Data Deficient), have a high extinction risk (42% of species occurring in the WIO are threatened), and are often range‐restricted (63% meet the range size threshold for Criterion B) (IUCN 2025). However, only one of the 19 electric ray species known from the WIO had sufficient data to meet the ISRA Criteria. Further, deepwater species are generally more isolated from human impacts (Dulvy et al. 2021), but their conservative life histories make them susceptible to rapid population declines when they are subject to anthropogenic threats (Finucci et al. 2024). Only 12% of deepwater species in the region were included in ISRAs, and almost all the available information was based on fisheries‐dependent methods such as fish‐market/landing‐site surveys and observers onboard commercial fishing vessels. Although fisheries are a threat to most of these species, they are also a key source of information on their local presence and abundance (Akhilesh et al. 2011; Everett et al. 2025) to delineate critical habitats (Fennessy et al. 2025; García‐Rodríguez et al. 2025). Increased use of fisheries‐dependent methods that incorporate spatial data on fishing locations could yield additional information on these species for relatively limited costs, although only in jurisdictions with active deepwater fisheries such as in Mozambique and India (Finucci et al. 2024). Scientific fishing surveys were the only fisheries‐independent method producing sufficient information to delineate ISRAs for the deep sea. Although more logistically complicated and expensive, this method could be used in areas that lack commercial fisheries. Non‐extractive deepwater methods were not used to delineate ISRAs in the WIO, but such methods are available and have been used to study deepwater chondrichthyans in the region. Examples include deep deployments of baited remote underwater video systems (Pearce et al. 2023), remotely operated vehicles (Frappi et al. 2023), and submersible dives (Garzon et al. 2022). Expanded use of these methods at sites of interest in the WIO, particularly in ABNJ, could yield valuable data to inform conservation efforts without requiring lethal sampling.

### 4.3 Geographic Coverage

Almost all jurisdictions hosted at least one ISRA, and there was no relationship between GDP and ISRA delineation. This suggests that information on chondrichthyans is being produced throughout the WIO region despite differences in national resources and capacity. This may result from the prevalence of accessible and inexpensive methods (e.g., visual census, fish‐market/landing‐site surveys, citizen science), multinational collaborations, or transboundary studies across multiple jurisdictions (Robinson et al. 2016; Daly et al. 2023), particularly those monitoring species movements (e.g., Berumen et al. 2014). Not surprisingly, most jurisdictions with larger EEZs and more diverse chondrichthyan assemblages have more ISRAs. Jordan was the only jurisdiction not to have an ISRA delineated in the region, which may be a result of its small EEZ (the second smallest in the region at 91 km^2^) within the chondrichthyan depauperate Red Sea (Compagno 1982). Further, only three ISRAs were delineated in ABNJ (although two additional ISRAs straddled a jurisdiction and international waters), reflecting the limited research undertaken in offshore waters. Despite this limited coverage, ISRAs delineated in the high seas offer a first opportunity to enhance conservation efforts for highly mobile species. These areas could serve as priority considerations at Regional Fisheries Management Organisations, particularly the Indian Ocean Tuna Commission, to assess appropriate fisheries management measures (e.g., gear restrictions, seasonal closures). Further, the Agreement under the United Nations Convention on the Law of the Sea on the Conservation and Sustainable Use of Marine Biological Diversity of Areas beyond National Jurisdiction (also known as the BBNJ Agreement) allows for the designation of MPAs or other areas‐based management tools in the high seas. ISRAs can be used to guide such designations toward critical chondrichthyan habitats in ABNJ (United Nations 2023).

The highest number of ISRAs was in the Maldives (27 ISRAs) and the Seychelles (11 ISRAs), archipelago nations with relatively large EEZs, high chondrichthyan biodiversity, valuable marine wildlife tourism sectors, and relatively high marine conservation attention. Conversely, fewer ISRAs were delineated in Mauritius, which has a large EEZ, a likely reflection of the limited dedicated research afforded to chondrichthyans as well as the type of data collated from existing projects. In the Maldives and Seychelles, the tourism sector has also invested significantly in both shark and ray tourism (Rowat and Engelhardt 2007; Anderson et al. 2011; Cagua et al. 2014; Zimmerhackel et al. 2019; Harvey‐Carroll et al. 2021), with visual census and citizen science conducted in association with local wildlife tourism operations providing much of the data used to delineate ISRAs. In the Maldives, many of these datasets can be traced to initiatives leveraging the local tourism industry for scientific data collection (Sattar et al. 2014). In jurisdictions without large marine tourism sectors, fisheries catch data tended to replace in situ observations as the main source of information. Examples include India (9 ISRAs), Iran (5 ISRAs), and Oman (5 ISRAs), where ISRA delineation was largely supported by information from fish market/landing site surveys, onboard observers, or scientific fishing.

Despite a broad review of the available information and delineating ~10% of the regional surface area as ISRAs, there was limited overlap with existing MPAs. With only 1.2% of ISRAs overlapping with no‐take MPAs, current spatial management is unlikely to make any meaningful contribution to the conservation and protection of chondrichthyans at the regional scale. This is not surprising given that MPAs in the WIO have not historically been designated with the protection of chondrichthyans in mind. The incidental nature of chondrichthyan protection under existing spatial management has resulted in the generally low overlap reported from the regions assessed to date, including the South and Central American Pacific (7% ISRA overlap with no‐take MPAs) (Mouton et al. 2024) and the Mediterranean and Black Seas (0.3% overlap) (Rohner et al. 2025).

The delineation of ISRAs presents an opportunity to align local chondrichthyan conservation efforts with global biodiversity targets. ISRAs can be integrated into national marine spatial planning and contribute to the design and creation of new MPAs. ISRAs can also be leveraged to identify sites for locally managed marine areas (LMMAs), which, with community support and policy alignment, can evolve into formally designated MPAs or other effective conservation measures. This bottom‐up pathway can be particularly effective in regions where community stewardship is strong but formal governance is still developing (e.g., Kenya, mainland Tanzania, and Madagascar) (Hattam et al. 2020). Incorporating the needs of chondrichthyans and other threatened taxa alongside other critical factors such as the presence of vulnerable marine ecosystems, commercial needs, and local cultural significance would maximize the potential benefits of spatial protections. Additionally, ISRA designations could help to expand the remits or ranges of existing MPAs to afford protection to chondrichthyans where needed (Faure‐Beaulieu et al. 2023; Mouton et al. 2024).

If implemented and enforced, such measures could have immediate conservation benefits for chondrichthyans while helping governments meet their commitments under the GBF. Within this context, and in recognition of ISRAs being a key tool to assist countries in meeting global biodiversity targets, Parties to the Convention on the Conservation of Migratory Species of Wild Animals (CMS; including 22 Parties from the WIO) and Signatories to its daughter agreement, the Sharks Memorandum of Understanding (Sharks MOU; including 11 Signatories from the WIO), passed several decisions related to ISRAs (CMS 2024). Specifically, CMS Parties and Signatories are requested to take into account identified ISRAs for spatial planning and conservation action, particularly for the benefit of CMS‐listed shark and ray species, while facilitating implementation of GBF Targets 1 and 3. The overlap analysis shows the high potential for some jurisdictions (particularly Amsterdam and Saint Paul Islands, Oman, and South Africa) to pursue their commitments under Target 3 by designating ISRAs as MPAs where appropriate.

The design and implementation of MPAs using ISRAs need to be approached from a pragmatic and evidence‐based perspective. The WIO is considered one of the worst basins in terms of illegal fisheries (Spijkers et al. 2023), and several no‐take MPAs in the region effectively serve as “paper parks” due to limited resources and enforcement capabilities (e.g., Collins et al. 2023). Enhancing spatial management frameworks and improving enforcement capacity are crucial to ensure these protections can have a positive impact on species. Further, many chondrichthyan species have movement ecologies or life histories that make them difficult to conserve using only area‐based management (Chin et al. 2023; Goetze et al. 2024). In these cases, transboundary cooperation will be key to success (e.g., Daly et al. 2023). Furthermore, alternative conservation strategies such as gear restrictions, size limits, or seasonal fishery closures could be used either independently or as complementary approaches (MacNeil et al. 2020).

Any protection will also be limited by human factors such as the capacity and political will for implementation and enforcement (Sethi and Hilborn 2008; Di Cintio et al. 2023). Indeed, many jurisdictions across the WIO already have protection or seasonal bans on fishing for several sharks and rays (Saudi Arabia, United Arab Emirates, Kuwait, Maldives [as a Shark Sanctuary]; Jabado and Spaet 2017). Still, sharks and rays continue to be landed as incidental catch because of limited enforcement (Jabado and Spaet 2017). Local stakeholders are also unlikely to tolerate severe disruptions to key economic activities such as fisheries, tourism, or shipping. Although we could not systematically assess overlap between delineated ISRAs and human industries with the available datasets, policy makers considering these sites for spatial protections will need to take such factors into account (Rohner et al. 2025). The ISRAs can be used to prioritise MPA designations where they are most needed, where they could be most effective for conserving critical habitats, and where they would be least disruptive to other uses (Rohner et al. 2025). At the regional scale of the WIO, the information derived from the ISRA process can inform systematic conservation planning for an ecologically coherent MPA network (Sundblad et al. 2011; Jonsson et al. 2020) that can now properly consider the needs of chondrichthyans alongside other priority taxa (Donald et al. 2019; Tetley et al. 2022; Wallace et al. 2023).

## 5 Conclusions

Chondrichthyans in the Indian Ocean have historically received less scientific research than those in the Atlantic or Pacific Oceans (Ducatez 2019). The WIO, in particular, is a known ‘dark spot’ for chondrichthyan conservation (Dulvy et al. 2024). By summarising available information and mapping critical habitats for chondrichthyans in the region, ISRAs provide a strategic framework for directing future research toward data‐poor species and regions while informing localised management strategies. This process was substantially strengthened by incorporating unpublished sources of data, much of which came from local wildlife tourism and fisheries operations. Strengthening collaboration with key stakeholders in these sectors could improve data collection, facilitate broader discussion and dissemination of results, and build trust with local communities throughout most jurisdictions. The diverse research methods used to support the different ISRA Criteria demonstrate the strengths of current data collection efforts and the potential for expanding those efforts to understudied species and offshore, deepwater habitats. However, the results also highlight key taxa and functional groups, as well as their geographic areas and habitats, that are systematically understudied and where novel approaches may be needed to fill persistent data gaps. Here, Areas of Interest in the WIO (Jabado et al. 2023) represent a low‐hanging fruit for future research and funding, particularly where local support and preliminary data already exist. The limited overlap between delineated ISRAs and existing MPAs underscores the insufficient protection afforded to critical chondrichthyan habitats. As such, ISRAs provide a powerful evidence base for WIO countries to expand these protections and meet their Target 3 commitments under the GBF while addressing vitally needed chondrichthyan conservation.

## Author Contributions

**Jesse E. M. Cochran:** conceptualization (equal), data curation (equal), formal analysis (equal), investigation (equal), methodology (equal), project administration (equal), visualization (equal), writing – original draft (lead). **Ryan Charles:** conceptualization (equal), data curation (equal), formal analysis (lead), investigation (equal), methodology (equal), visualization (equal), writing – original draft (equal). **Andrew J. Temple:** conceptualization (equal), data curation (equal), formal analysis (lead), investigation (equal), methodology (equal), visualization (equal), writing – original draft (equal). **Peter M. Kyne:** conceptualization (equal), data curation (equal), investigation (equal), writing – original draft (equal), writing – review and editing (lead). **Emiliano García‐Rodríguez:** conceptualization (equal), data curation (equal), investigation (equal), writing – review and editing (equal). **Adriana Gonzalez‐Pestana:** conceptualization (equal), data curation (equal), investigation (equal), writing – review and editing (equal). **Amanda Batlle‐Morera:** conceptualization (equal), data curation (equal), investigation (equal), writing – review and editing (equal). **Théophile L. Mouton:** conceptualization (equal), data curation (equal), investigation (equal), writing – review and editing (equal). **Asia O. Armstrong:** conceptualization (equal), data curation (equal), investigation (equal), writing – review and editing (equal). **Christoph A. Rohner:** conceptualization (equal), data curation (equal), investigation (equal), writing – review and editing (equal). **Darren J. Coker:** conceptualization (equal), data curation (equal), investigation (equal), writing – review and editing (equal). **Royale S. Hardenstine:** conceptualization (equal), data curation (equal), investigation (equal), writing – review and editing (equal). **Alexander Kattan:** conceptualization (equal), data curation (equal), investigation (equal), writing – review and editing (equal). **Ashlie J. McIvor:** conceptualization (equal), data curation (equal), investigation (equal), writing – review and editing (equal). **Viktor Nunes Peinemann:** conceptualization (equal), data curation (equal), investigation (equal), writing – review and editing (equal). **Kaitlyn A. O'Toole:** conceptualization (equal), data curation (equal), investigation (equal), writing – review and editing (equal). **Lea Palm:** conceptualization (equal), data curation (equal), investigation (equal), writing – review and editing (equal). **Eloise B. Richardson:** conceptualization (equal), data curation (equal), investigation (equal), writing – review and editing (equal). **Kalli Valappil Akhilesh:** data curation (equal), investigation (equal), writing – review and editing (equal). **Haleh Ali Abedi:** data curation (equal), investigation (equal), writing – review and editing (equal). **Reem K. Almealla:** data curation (equal), investigation (equal), writing – review and editing (equal). **Dareen Almojil:** data curation (equal), investigation (equal), writing – review and editing (equal). **Samantha Andrzejaczek:** data curation (equal), investigation (equal), writing – review and editing (equal). **Arzucan N. Askin:** data curation (equal), investigation (equal), writing – review and editing (equal). **Avik A. Banerjee:** data curation (equal), investigation (equal), writing – review and editing (equal). **Hamid R. Bargahi:** data curation (equal), investigation (equal), writing – review and editing (equal). **Alissa J. Barnes:** data curation (equal), investigation (equal), writing – review and editing (equal). **Svetlana Barteneva‐Vitry:** data curation (equal), investigation (equal), writing – review and editing (equal). **Siamak Behzadi:** data curation (equal), investigation (equal), writing – review and editing (equal). **Aymeric Bein:** data curation (equal), investigation (equal), writing – review and editing (equal). **Rhett H. Bennett:** data curation (equal), investigation (equal), writing – review and editing (equal). **Filippo Bocchi:** data curation (equal), funding acquisition (equal), writing – review and editing (equal). **Ginevra Boldrocchi:** data curation (equal), investigation (equal), writing – review and editing (equal). **Gill T. Braulik:** data curation (equal), investigation (equal), writing – review and editing (equal). **Camrin D. Braun:** data curation (equal), investigation (equal), writing – review and editing (equal). **Eleanor Brighton:** data curation (equal), investigation (equal), writing – review and editing (equal). **Frances K. P. Budd:** data curation (equal), investigation (equal), writing – review and editing (equal). **Robert W. Bullock:** data curation (equal), investigation (equal), writing – review and editing (equal). **Clara Canovas Perez:** data curation (equal), investigation (equal), writing – review and editing (equal). **Aaron B. Carlisle:** data curation (equal), investigation (equal), writing – review and editing (equal). **Michelle Carpenter:** data curation (equal), investigation (equal), writing – review and editing (equal). **Taylor K. Chapple:** data curation (equal), investigation (equal), writing – review and editing (equal). **Isabel Chaúca:** data curation (equal), investigation (equal), writing – review and editing (equal). **Geremy Cliff:** data curation (equal), investigation (equal), writing – review and editing (equal). **Estelle Crochelet:** data curation (equal), investigation (equal), writing – review and editing (equal). **Nakia Cullain:** data curation (equal), investigation (equal), writing – review and editing (equal). **David J. Curnick:** data curation (equal), investigation (equal), writing – review and editing (equal). **Ryan Daly:** data curation (equal), investigation (equal), writing – review and editing (equal). **Leigh de Necker:** data curation (equal), investigation (equal), writing – review and editing (equal). **Stella Diamant:** data curation (equal), investigation (equal), writing – review and editing (equal). **Giulia F. A. Donati:** data curation (equal), investigation (equal), writing – review and editing (equal). **David A. Ebert:** data curation (equal), investigation (equal), writing – review and editing (equal). **Ehab Eid:** data curation (equal), investigation (equal), writing – review and editing (equal). **Igbal S. Elhassa:** data curation (equal), investigation (equal), writing – review and editing (equal). **Chantel Elston:** data curation (equal), investigation (equal), writing – review and editing (equal). **Bernadine I. Everett:** data curation (equal), investigation (equal), writing – review and editing (equal). **Mahmoud M. S. Farrag:** data curation (equal), investigation (equal), writing – review and editing (equal). **Nico Fassbender:** data curation (equal), investigation (equal), writing – review and editing (equal). **Sean T. Fennessy:** data curation (equal), investigation (equal), writing – review and editing (equal). **Stela M. C. Fernando:** data curation (equal), investigation (equal), writing – review and editing (equal). **Brittany Finucci:** data curation (equal), investigation (equal), writing – review and editing (equal). **Anna L. Flam:** data curation (equal), investigation (equal), writing – review and editing (equal). **Peter Gausman:** data curation (equal), investigation (equal), writing – review and editing (equal). **Arnault R. G. Gauthier:** data curation (equal), investigation (equal), writing – review and editing (equal). **Giri Bhavan Sreekanth:** conceptualization (equal), investigation (equal), writing – review and editing (equal). **Trisha Gupta:** data curation (equal), investigation (equal), writing – review and editing (equal). **Meral Hafeez:** data curation (equal), investigation (equal), writing – review and editing (equal). **Badrú N. Hagy:** data curation (equal), investigation (equal), writing – review and editing (equal). **Jessica L. A. Haines:** data curation (equal), investigation (equal), writing – review and editing (equal). **Joanna L. Harris:** data curation (equal), investigation (equal), writing – review and editing (equal). **Jessica Harvey‐Carroll:** data curation (equal), investigation (equal), writing – review and editing (equal). **Tessa N. Hempson:** data curation (equal), investigation (equal), writing – review and editing (equal). **Simon T. Hilbourne:** data curation (equal), investigation (equal), writing – review and editing (equal). **Hua Hsun Hsu:** data curation (equal), investigation (equal), writing – review and editing (equal). **Nor D. Ibrahim:** data curation (equal), investigation (equal), writing – review and editing (equal). **David M. P. Jacoby:** data curation (equal), investigation (equal), writing – review and editing (equal). **Sébastien Jaquemet:** data curation (equal), investigation (equal), writing – review and editing (equal). **Idrees Babu K K:** data curation (equal), investigation (equal), writing – review and editing (equal). **Divya Karnad:** data curation (equal), investigation (equal), writing – review and editing (equal). **Boaz Kaunda‐Arara:** data curation (equal), investigation (equal), writing – review and editing (equal). **Shoba J. Kizhakudan:** data curation (equal), investigation (equal), writing – review and editing (equal). **Alison A. Kock:** data curation (equal), investigation (equal), writing – review and editing (equal). **Anna Koester:** data curation (equal), investigation (equal), writing – review and editing (equal). **Bigeyo N. Kuboja:** data curation (equal), investigation (equal), writing – review and editing (equal). **Baraka L. Kuguru:** data curation (equal), investigation (equal), writing – review and editing (equal). **James S. E. Lea:** data curation (equal), investigation (equal), writing – review and editing (equal). **Omar Mahadalle:** data curation (equal), investigation (equal), writing – review and editing (equal). **Hashim Manjebrayakath:** data curation (equal), investigation (equal), writing – review and editing (equal). **Christophe Mason‐Parker:** data curation (equal), investigation (equal), writing – review and editing (equal). **Daniel Mateos‐Molina:** data curation (equal), investigation (equal), writing – review and editing (equal). **Muktha Menon:** data curation (equal), investigation (equal), writing – review and editing (equal). **Alec B. M. Moore:** data curation (equal), investigation (equal), writing – review and editing (equal). **Johann Mourier:** data curation (equal), investigation (equal), writing – review and editing (equal). **Taryn S. Murra:** data curation (equal), investigation (equal), writing – review and editing (equal). **Ajay D. Nakhawa:** data curation (equal), investigation (equal), writing – review and editing (equal). **Nadeem Nazurally:** data curation (equal), investigation (equal), writing – review and editing (equal). **Lauren E. Nelso:** data curation (equal), investigation (equal), writing – review and editing (equal). **John E. G. Nevill:** data curation (equal), investigation (equal), writing – review and editing (equal). **Jennifer M. Olbers:** data curation (equal), investigation (equal), writing – review and editing (equal). **Raquel L. Ostrovski:** data curation (equal), investigation (equal), writing – review and editing (equal). **Lauren R. Peel:** data curation (equal), investigation (equal), writing – review and editing (equal). **Nathan Perisic:** data curation (equal), investigation (equal), writing – review and editing (equal). **Bradley Peterson:** data curation (equal), investigation (equal), writing – review and editing (equal). **Simon J. Pierce:** data curation (equal), investigation (equal), writing – review and editing (equal). **Simon J. Pittman:** data curation (equal), investigation (equal), writing – review and editing (equal). **Shikha Rahangdale:** data curation (equal), investigation (equal), writing – review and editing (equal). **Joshua Rambahiniarison:** data curation (equal), investigation (equal), writing – review and editing (equal). **Ali Reza Rastgoo:** data curation (equal), investigation (equal), writing – review and editing (equal). **Mohsen Rezaie‐Atagholipour:** data curation (equal), investigation (equal), writing – review and editing (equal). **David P. Robinson:** data curation (equal), investigation (equal), writing – review and editing (equal). **Melita A. Samoilys:** data curation (equal), investigation (equal), writing – review and editing (equal). **Tamaryn J. Sawers:** data curation (equal), investigation (equal), writing – review and editing (equal). **Brittney J. Scannell:** data curation (equal), investigation (equal), writing – review and editing (equal). **Jennifer V. Schmidt:** data curation (equal), investigation (equal), writing – review and editing (equal). **Isabel M. Silva:** data curation (equal), investigation (equal), writing – review and editing (equal). **Luis Silva:** data curation (equal), investigation (equal), writing – review and editing (equal). **Jadiyde Solonomenjanahary:** data curation (equal), investigation (equal), writing – review and editing (equal). **Julia L. Y. Spaet:** data curation (equal), investigation (equal), writing – review and editing (equal). **Guy M. W. Stevens:** data curation (equal), investigation (equal), writing – review and editing (equal). **Elspeth M. Strike:** data curation (equal), investigation (equal), writing – review and editing (equal). **Sujitha Thomas:** data curation (equal), investigation (equal), writing – review and editing (equal). **David van Beuningen:** data curation (equal), investigation (equal), writing – review and editing (equal). **Stephanie K. Venables:** data curation (equal), investigation (equal), writing – review and editing (equal). **Lennart Vossgaetter:** data curation (equal), investigation (equal), writing – review and editing (equal). **Ornella C. Weideli:** data curation (equal), investigation (equal), writing – review and editing (equal). **Ivor D. Williams:** conceptualization (equal), investigation (equal), writing – review and editing (equal). **Collin T. Williams:** data curation (equal), investigation (equal), writing – review and editing (equal). **Andrew J. Willson:** data curation (equal), investigation (equal), writing – review and editing (equal). **Livi Wilson:** data curation (equal), investigation (equal), writing – review and editing (equal). **Irthisham H. Zareer:** data curation (equal), investigation (equal), writing – review and editing (equal). **Kaitlyn M. Zerr:** data curation (equal), investigation (equal), writing – review and editing (equal). **Michael L. Berumen:** data curation (equal), funding acquisition (supporting), investigation (equal), supervision (equal), writing – review and editing (equal). **Rima W. Jabado:** conceptualization (equal), data curation (equal), funding acquisition (lead), investigation (equal), project administration (lead), supervision (equal), writing – original draft (equal), writing – review and editing (equal).

## Conflicts of Interest

The authors declare no conflicts of interest.

## Supporting information


**Appendix S1:** ece372690‐sup‐0001‐supinfo.docx.

**Data S1:** ece372690‐sup‐0002‐DataS1.xlsx.

## Data Availability

The complete information for species, habitats, and ISRA Criteria related to each one of the ISRAs can be found and downloaded from the ISRA website (https://sharkrayareas.org/). IUCN distribution maps are available on the IUCN Red List website (https://www.iucnredlist.org/).
