# Correction to “Only One Percent of Important Shark and Ray Areas in the Western Indian Ocean Are Fully Protected From Fishing Pressure”

**DOI:** 10.1002/ece3.73121

**Published:** 2026-02-17

Cochran, J.E.M., Charles, R., Temple, A.J., Kyne, P.M., García‐Rodríguez, E., Gonzalez‐Pestana, A., Batlle‐Morera, A., Mouton, T.L., Armstrong, A.O., Rohner, C.A., Coker, D.J., Hardenstine, R.S., Kattan, A., McIvor, A.J., Peinemann, V.N., O'Toole, K.A., Palm, L., Richardson, E.B., Akhilesh, K.V., Ali Abedi, H., Almealla, R.K., Almojil, D., Andrzejaczek, S., Askin, A.N., Banerjee, A.A., Bargahi, H.R., Barnes, A.J., Barteneva‐Vitry, S., Behzadi, S., Bein, A., Bennett, R.H., Bocchi, F., Boldrocchi, G., Braulik, G.T., Braun, C.D., Brighton, E., Budd, F.K.P., Bullock, R.W., Canovas Perez, C., Carlisle, A.B., Carpenter, M., Chapple, T.K., Chaúca, I., Cliff, G., Crochelet, E., Cullain, N., Curnick, D.J., Daly, R., de Necker, L., Diamant, S., Donati, G.F.A., Ebert, D.A., Eid, E., Elhassan, I.S., Elston, C., Everett, B.I., Farrag, M.M.S., Fassbender, N., Fennessy, S.T., Fernando, S.M.C., Finucci, B., Flam, A.L., Gausmann, P., Gauthier, A.R.G., Sreekanth, G.B., Gupta, T., Hafeez, M., Hagy, B.N., Haines, J.L.A., Harris, J.L., Harvey‐Carroll, J., Hempson, T.N., Hilbourne, S.T., Hsu, H.H., Ibrahim, N.D., Jacoby, D.M.P., Jaquemet, S., Babu K K, I., Karnad, D., Kaunda‐Arara, B., Kizhakudan, S.J., Kock, A.A., Koester, A., Kuboja, B.N., Kuguru, B.L., Lea, J.S.E., Mahadalle, O., Manjebrayakath, H., Mason‐Parker, C., Mateos‐Molina, D., Menon, M., Moore, A.B.M., Mourier, J., Murray, T.S., Nakhawa, A.D., Nazurally, N., Nelson, L.E., Nevill, J.E.G., Olbers, J.M., Ostrovski, R.L., Peel, L.R., Perisic, N., Peterson, B., Pierce, S.J., Pittman, S.J., Rahangdale, S., Rambahiniarison, J., Rastgoo, A.R., Rezaie‐Atagholipour, M., Robinson, D.P., Samoilys, M.A., Sawers, T.J., Scannell, B.J., Schmidt, J.V., Silva, I.M., Silva, L., Solonomenjanahary, J., Spaet, J.L.Y., Stevens, G.M.W., Strike, E.M., Thomas, S., van Beuningen, D., Venables, S.K., Vossgaetter, L., Weideli, O.C., Williams, I.D., Williams, C.T., Willson, A.J., Wilson, L., Zareer, I.H., Zerr, K.M., Berumen, M.L. and Jabado, R.W. (2026), Only One Percent of Important Shark and Ray Areas in the Western Indian Ocean Are Fully Protected From Fishing Pressure. *Ecology and Evolution*, 16: e72690. https://doi.org/10.1002/ece3.72690

The surnames of the following authors were incorrect in the published article. The complete names are as follows:

1. Peter Gausmann
2. Taryn S. Murray
3. Igbal S. Elhassan
4. Lauren E. Nelson

The online version of this article has been corrected accordingly.

We apologize for this error.

