# Supplementary material for: Only One Percent of Important Shark and Ray Areas in the Western Indian Ocean Are Fully Protected From Fishing Pressure
Source: Ecol Evol. 2026 Jan 11;16(1):e72690. doi: 10.1002/ece3.72690 (PMC12793059; doi:10.1002/ece3.72690)
Supplement: Supplementary file 1 — Appendix S1: ece372690‐sup‐0001‐supinfo.docx. [file ECE3-16-e72690-s002.docx]

SUPPLEMENTARY INFORMATION


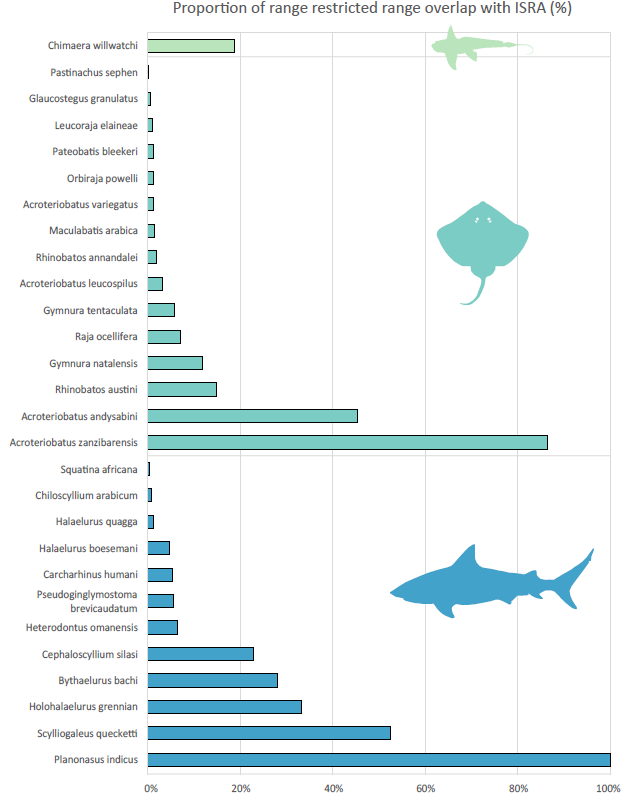


**SI Figure 1.** The proportion of overlap between the global geographic range for each Range Restricted Qualifying Species and the Important Shark and Ray Area(s) in the Western Indian Ocean. The geographic range of each species is sourced from the IUCN Red List of Threatened Species (IUCN 2025).

**SI Table 1.** Categories and respective definitions of research methods used to obtain data to support the delineation of Important Shark and Ray Areas in the Western Indian Ocean.

| **Research Method Category** | **Definition** |
| --- | --- |
| Scientific Fishing | Fisheries-independent catch records from fishing effort conducted by researchers specifically for specimen collections or to record catch-per-unit-effort. This can include scientific longlines, trawls, or any other gear type. Catch and release fishing for other methods like electronic tagging, should not be included under this method. |
| Fish-Market/Landing-Site Surveys | Fisheries-dependent landing records and specimen collections taken by researchers from shore, either at a fish market or landing site. May include records or specimens from artisanal, commercial, or recreational fisheries. |
| Fisheries Observer Data/Logbooks | Fisheries-dependent catch records taken by dedicated observers or logged by crew onboard commercial fishing vessels. Distinct from landings records in that all catches can be recorded (e.g., discards). |
| Citizen Science | Data collected in a volunteer capacity by the wider public. This can take many forms but usually consists of video or photographic records from recreational diving or fishing activities. This method is often used to supplement more traditional datasets maintained by researchers. If non-researchers originally collected a substantial portion of the data, Citizen Science should be listed as one of the methods used. |
| Local Ecological Knowledge | Anecdotal or historical data collected from local community members. These data are often used to guide research efforts and can be invaluable. Still, they need to be verified by physical evidence (usually photos or videos) or confirmed using other research methods. If researchers were originally advised or guided to a site by local contacts, Local Ecological Knowledge should be included as one of the methods used. |
| Informal Researcher Observations | Random or opportunistic records that are not collected as part of a dedicated study or survey. Data cited as “personal observation” is listed under this method. |
| Electronic Tracking | Movement data recorded using acoustic or satellite telemetry. Most often used to delineate Movement Areas (Sub-criterion C4) but can also be used to define Reproductive Areas (Sub-criterion C1) and other critical habitats. |
| Mark-recapture | Encounter records collected using simple number tags or photos of an animal’s natural markings to distinguish individuals. |
| Visual Census | Visual census conducted as part of a structured survey either at the surface (shore-based or boat-based surveys) or *in-situ* (diver or snorkeler surveys) and may be assisted with photography, videography (including Diver Operated Video Systems), or other data collection tools. |
| Remote Video | Footage collection using camera traps (baited or unbaited) or animal-borne cameras. Most often through the deployment of Baited Remote Underwater Video Stations (BRUVS). |
| Aerial Surveys | Surveys using aerial platforms including unmanned aerial vehicles (UAVs), helicopters, and microlites. |
| Biochemical Analysis | Stable isotopes, fatty acids, or other biochemical markers used to support foraging behavior for Feeding Areas (Sub-criterion C2). |

**SI Table 2:** Hurdle model outputs for species-specific influences on the probability and frequency of ISRA delineation. Adjusted p-values reflect the results of a Holm-Bonferroni correction to account for multiple comparisons. Both raw p-values and the more conservative adjusted p-values are highlighted in green when they would suggest a statistically significant relationship between a given influence and ISRA delineation/frequency.

**Species Hurdle Models**

|  | **Estimate** | **Std.Error** | **Z-value** | ***p*-value** | **Adjusted *p*** |
| --- | --- | --- | --- | --- | --- |
| **Median Depth, *n = 801, residual df = 792, AIC = 1766.2*** | | | | | |
| ***Hurdle (0/1), R^2^ = 0.105*** | | | | | |
| **Pub** | **1.3471** | **0.1612** | **8.354** | **<0.001** | **<0.001** |
| **Pub + Mixed** | **0.974** | **1.44E-01** | **6.747** | **<0.001** | **<0.001** |
| **All** | **0.9063** | **1.42E-01** | **6.373** | **<0.001** | **<0.001** |
| ***Count (conditional on inclusion), R^2^ = 0.065*** | | | | | |
| **Pub** | **0.278** | **0.2594** | **1.072** | **0.284** | **0.284** |
| **Pub + Mixed** | **0.7311** | **0.2631** | **2.778** | **0.005** | **0.011** |
| **All** | **1.1123** | **0.2844** | **3.911** | **<0.001** | **<0.001** |
| **Maximum Linear Dimension, *n = 801, residual df = 792, AIC = 1623.2*** | | | | | |
| ***Hurdle (0/1), R^2^ = 0.183*** | | | | | |
| **Pub** | **-2.1792** | **0.2472** | **-8.817** | **<0.001** | **<0.001** |
| **Pub + Mixed** | **-2.5782** | **0.2544** | **-10.134** | **<0.001** | **<0.001** |
| **All** | **-2.6554** | **0.2566** | **-10.349** | **<0.001** | **<0.001** |
| ***Count (conditional on inclusion), R^2^ = 0.155*** | | | | | |
| **Pub** | **2.4691** | **0.3802** | **6.494** | **<0.001** | **<0.001** |
| **Pub + Mixed** | **2.981** | **0.401** | **7.434** | **<0.001** | **<0.001** |
| **All** | **3.3217** | **0.4137** | **8.03** | **<0.001** | **<0.001** |
| **Species Range, *n = 774, residual df = 765, AIC = 1612.5*** | | | | | |
| ***Hurdle (0/1), R^2^ = 0.207*** | | | | | |
| **Pub** | **-0.81268** | **0.09609** | **-8.458** | **<0.001** | **<0.001** |
| **Pub + Mixed** | **-0.97547** | **0.09912** | **-9.841** | **<0.001** | **<0.001** |
| **All** | **-1.00945** | **0.10001** | **-10.094** | **<0.001** | **<0.001** |
| ***Count (conditional on inclusion), R^2^ = 0.050*** | | | | | |
| **Pub** | **0.9254** | **0.1754** | **5.276** | **<0.001** | **<0.001** |
| **Pub + Mixed** | **1.1138** | **0.1809** | **6.158** | **<0.001** | **<0.001** |
| **All** | **1.2525** | **0.1868** | **6.704** | **<0.001** | **<0.001** |

**SI Table 3:** Post-hoc analysis comparing results among the species hurdle models fitted with different levels of inclusion for the unpublished information. Adjusted p-values reflect the results of a Holm-Bonferroni correction to account for multiple comparisons. Both raw p-values and the more conservative adjusted p-values are highlighted in green when they would suggest a statistically significant difference in outputs between a given pair of models.

**Species post-hoc analyses**

|  | **Estimate** | **Std.Error** | **Z-ratio** | ***p*-value** | **Adjusted *p*** |
| --- | --- | --- | --- | --- | --- |
| **Median Depth** | | | | | |
| ***Hurdle (0/1)*** | | | | | |
| **Pub/Mixed** | **0.3731** | **0.0985** | **3.789** | **<0.001** | **0.002** |
| **Pub/All** | **0.4408** | **0.0974** | **4.526** | **<0.001** | **0.002** |
| **Mixed/All** | **0.0677** | **0.0835** | **0.811** | **0.417** | **1.000** |
| ***Count (conditional on inclusion)*** | | | | | |
| **Pub/Mixed** | **-0.453** | **0.168** | **-2.701** | **0.007** | **0.035** |
| **Pub/All** | **-0.834** | **0.174** | **-4.808** | **<0.001** | **0.002** |
| **Mixed/All** | **-0.381** | **0.147** | **-2.596** | **0.009** | **0.038** |
| **Maximum Linear Dimension** | | | | | |
| ***Hurdle (0/1)*** | | | | | |
| **Pub/Mixed** | **0.399** | **0.0995** | **4.01** | **<0.001** | **0.002** |
| **Pub/All** | **0.4762** | **0.0996** | **4.782** | **<0.001** | **0.002** |
| **Mixed/All** | **0.0773** | **0.094** | **0.822** | **0.411** | **1.000** |
| ***Count (conditional on inclusion)*** | | | | | |
| **Pub/Mixed** | **-0.512** | **0.142** | **-3.617** | **<0.001** | **0.003** |
| **Pub/All** | **-0.853** | **0.143** | **-5.951** | **<0.001** | **0.002** |
| **Mixed/All** | **-0.341** | **0.11** | **-3.099** | **0.002** | **0.013** |
| **Species Range** | | | | | |
| ***Hurdle (0/1)*** | | | | | |
| **Pub/Mixed** | **0.163** | **0.0398** | **4.09** | **<0.001** | **0.002** |
| **Pub/All** | **0.197** | **0.0399** | **4.934** | **<0.001** | **0.002** |
| **Mixed/All** | **0.034** | **0.0378** | **0.899** | **0.368** | **1.000** |
| ***Count (conditional on inclusion)*** | | | | | |
| **Pub/Mixed** | **-0.188** | **0.0595** | **-3.166** | **0.002** | **0.012** |
| **Pub/All** | **-0.327** | **0.0604** | **-5.414** | **<0.001** | **0.002** |
| **Mixed/All** | **-0.139** | **0.0481** | **-2.883** | **0.004** | **0.023** |

**SI Table 4:** Hurdle model outputs for jurisdiction-specific influences on the probability and frequency of ISRA delineation. Adjusted p-values reflect the results of a Holm-Bonferroni correction to account for multiple comparisons. Both raw p-values and the more conservative adjusted p-values are highlighted in green when they would suggest a statistically significant relationship between a given influence and ISRA delineation/frequency.

**Jurisdictional Hurdle Models**

|  | **Estimate** | **Std.Error** | **Z-value** | ***p*-value** | **Adjusted *p*** |
| --- | --- | --- | --- | --- | --- |
| **GDP, *n = 81, residual df = 72, AIC = 393.5*** | | | | | |
| ***Hurdle (0/1), R^2^ = 0.132*** | | | | | |
| **Pub** | **-0.2671** | **0.3435** | **-0.778** | **0.437** | **1.000** |
| **Pub + Mixed** | **-0.8749** | **0.4596** | **-1.904** | **0.057** | **0.342** |
| **All** | **-1.6719** | **0.7931** | **-2.108** | **0.035** | **0.245** |
| ***Count (conditional on inclusion), R^2^ = 0.106*** | | | | | |
| **Pub** | **-0.3696** | **0.2022** | **-1.828** | **0.068** | **0.342** |
| **Pub + Mixed** | **-0.1064** | **0.191** | **-0.557** | **0.577** | **1.000** |
| **All** | **0.1336** | **0.1993** | **0.67** | **0.503** | **1.000** |
| **Species Richness, *n = 87, residual df = 78, AIC = 391.3*** | | | | | |
| ***Hurdle (0/1), R^2^ = 0.263*** | | | | | |
| **Pub** | **-6.022** | **2.199** | **-2.738** | **0.006** | **0.068** |
| **Pub + Mixed** | **-6.932** | **2.305** | **-3.008** | **0.003** | **0.034** |
| **All** | **-7.914** | **2.486** | **-3.184** | **0.001** | **0.020** |
| ***Count (conditional on inclusion), R^2^ = 0.218*** | | | | | |
| **Pub** | **2.1179** | **0.8435** | **2.511** | **0.012** | **0.108** |
| **Pub + Mixed** | **2.5502** | **0.8681** | **2.938** | **0.003** | **0.040** |
| **All** | **2.9284** | **0.8961** | **3.268** | **0.001** | **0.016** |
| **EEZ Area, *n = 87, residual df = 78, AIC = 369.6*** | | | | | |
| ***Hurdle (0/1), R^2^ = 0.240*** | | | | | |
| **Pub** | **-0.4095** | **0.2326** | **-1.76** | **0.078** | **0.342** |
| **Pub + Mixed** | **-0.7449** | **0.2735** | **-2.724** | **0.006** | **0.068** |
| **All** | **-1.3602** | **0.5806** | **-2.343** | **0.019** | **0.153** |
| ***Count (conditional on inclusion), R^2^ = 0.255*** | | | | | |
| **Pub** | **0.8993** | **0.2065** | **4.355** | **<0.001** | **<0.001** |
| **Pub + Mixed** | **1.0266** | **0.2062** | **4.978** | **<0.001** | **<0.001** |
| **All** | **1.1338** | **0.2082** | **5.447** | **<0.001** | **<0.001** |

**SI Table 5:** Post-hoc analysis comparing results among the jurisdiction hurdle models fitted with different levels of inclusion for the unpublished information. Adjusted p-values reflect the results of a Holm-Bonferroni correction to account for multiple comparisons. Both raw p-values and the more conservative adjusted p-values are highlighted in green when they would suggest a statistically significant difference in outputs between a given pair of models.

**Jurisdictional post-hoc analyses**

|  | **Estimate** | **Std.Error** | **Z-ratio** | ***p*-value** | **Adjusted *p*** |
| --- | --- | --- | --- | --- | --- |
| **GDP** | | | | | |
| ***Hurdle (0/1)*** | | | | | |
| **Pub/Mixed** | **0.608** | **0.399** | **1.522** | **0.128** | **0.767** |
| **Pub/All** | **1.405** | **0.725** | **1.937** | **0.053** | **0.379** |
| **Mixed/All** | **0.797** | **0.765** | **1.041** | **0.298** | **0.893** |
| ***Count (conditional on inclusion)*** | | | | | |
| **Pub/Mixed** | **-0.263** | **0.199** | **-1.321** | **0.187** | **0.893** |
| **Pub/All** | **-0.503** | **0.199** | **-2.526** | **0.012** | **0.173** |
| **Mixed/All** | **-0.24** | **0.181** | **-1.329** | **0.184** | **0.893** |
| **Species Richness** | | | | | |
| ***Hurdle (0/1)*** | | | | | |
| **Pub/Mixed** | **0.91** | **0.414** | **2.197** | **0.028** | **0.336** |
| **Pub/All** | **1.893** | **0.71** | **2.666** | **0.008** | **0.123** |
| **Mixed/All** | **0.983** | **0.731** | **1.345** | **0.179** | **0.893** |
| ***Count (conditional on inclusion)*** | | | | | |
| **Pub/Mixed** | **-0.432** | **0.202** | **-2.136** | **0.033** | **0.336** |
| **Pub/All** | **-0.81** | **0.205** | **-3.947** | **0.000** | **0.002** |
| **Mixed/All** | **-0.378** | **0.167** | **-2.262** | **0.024** | **0.332** |
| **EEZ Area** | | | | | |
| ***Hurdle (0/1)*** | | | | | |
| **Pub/Mixed** | **0.335** | **0.153** | **2.194** | **0.028** | **0.336** |
| **Pub/All** | **0.951** | **0.48** | **1.982** | **0.047** | **0.379** |
| **Mixed/All** | **0.615** | **0.485** | **1.268** | **0.205** | **0.893** |
| ***Count (conditional on inclusion)*** | | | | | |
| **Pub/Mixed** | **-0.127** | **0.0623** | **-2.042** | **0.041** | **0.370** |
| **Pub/All** | **-0.234** | **0.0602** | **-3.894** | **0.000** | **0.002** |
| **Mixed/All** | **-0.107** | **0.048** | **-2.232** | **0.026** | **0.333** |
